# The location and development of Replicon Cluster Domains in early replicating DNA

**DOI:** 10.12688/wellcomeopenres.18742.1

**Published:** 2023-04-11

**Authors:** José A. da Costa-Nunes, Marek Gierlinski, Takayo Sasaki, Emma J. Haagensen, David M. Gilbert, J. Julian Blow

**Affiliations:** 1 Centre for Gene Regulation & Expression, School of Life Sciences, University of Dundee, Dundee, DD1 5EH, UK; 2 Data Analysis Group, School of Life Sciences, University of Dundee, Dundee, DD1 5EH, UK; 3 San Diego Biomedical Research Institute, San Diego, California, CA 92121, USA; 4 Present address: School of Medical Education, Faculty of Medical Sciences, Newcastle University, Newcastle upon Tyne, NE2 4HH, UK

**Keywords:** DNA replication, S phase, replicon clusters, replication timing, cell cycle

## Abstract

**Background**: It has been known for many years that in metazoan cells, replication origins are organised into clusters where origins within each cluster fire near-synchronously. Despite clusters being a fundamental organising principle of metazoan DNA replication, the location of origin clusters on the genome has not been documented.

**Methods**: We synchronised human U2OS by thymidine block and release followed by a brief block with L-mimosine to create a population of cells progressing into S phase with a high degree of synchrony. At different times after release into S phase, cells were pulsed with EdU; the EdU-labelled DNA was then pulled down, sequenced and mapped back onto the human genome.

**Results**: The early replicating DNA showed features at a range of scales. Wavelet analysis showed that the major feature of the early replicating DNA was at a size of 500 kb, consistent with clusters of replication origins. Over the first two hours of S phase, these Replicon Cluster Domains broadened in width, consistent with their being enlarged by the progression of replication forks at their outer boundaries. The total replication signal associated with each Replicon Cluster Domain varied considerably, and this variation was reproducible and conserved over time. We provide evidence that this variability in replication signal was at least in part caused by Replicon Cluster Domains being activated at different times in different cells in the population. We also provide evidence that adjacent clusters were preferentially activated in sequence across a group, consistent with the ‘domino’ model of replication focus activation observed by microscopy.

**Conclusions**: We show that early replicating DNA is organised into Replicon Cluster Domains that behave as expected of replicon clusters observed by DNA fibre analysis. The coordinated activation of different Replicon Cluster Domains can generate the replication timing programme by which the genome is duplicated.

## Introduction

The human genome harbours approximately 6.6 Gbp of DNA that is split between 46 chromosomes ^1^ and is packed in the nucleus in chromatin bearing different degrees of compaction ^2^. Almost all of the DNA is replicated during S phase of the cell division cycle. In somatic human cells, S phase typically lasts around 10 hours, and the full cell cycle lasts approximately 24 hours ^3, 4^.

In late mitosis and G1, prior to the initiation of DNA replication in S phase, the sites of where replication initiation can take place (DNA replication origins) are licensed by being encircled by double hexamers of the MCM2-7 proteins. However, more DNA replication origins are licensed in G1 than are activated during S phase ^5^. Indeed, of the total number of potentially available replication origins present in diploid mammalian cells – roughly 100,000,000 – only approximately 10% will be used to initiate DNA replication ^3, 5– 7^. The segment of DNA replicated by the advancement of the fork or forks emanating from one origin is called a replicon and has an average size of ~150 Kbp ^4, 7– 9^. Given a fork speed of 1–2 kb/min ^8, 10, 11^ an average replicon is therefore active for 37.5–75 min. Some ‘strong’ replication origins are active in almost all cells in a population, but for weaker origins there is a high degree of cell-to-cell variability in exactly which origins fire and which remain dormant ^7, 12– 14^.

Extensive data from DNA fibre analysis has shown that replication origins are typically organised into clusters of two to ten origins (typically from three to six) that fire near-synchronously during S phase ^8, 15^. The average size of an origin cluster would therefore be in the range of 400–800 kb. The clustering of initiation events at particular loci may be a consequence of origins with similar replication timing being grouped together in regions ^7^. A recent paper has provided evidence that replication initiation domains are characterized by highly efficient origins flanking a cluster of less efficient origins ^14^. However, to date, there is little understanding of the actual DNA sequences that make up origin clusters.

At a higher level, it is observed that large (megabase) regions of DNA replicate at characteristic times in S phase; these regions are termed ‘replication timing domains’ (RTDs) ^16– 21^. With the advent of next generation sequencing, it became possible to identify the different timing domains and correlate them with higher-order nuclear architecture ^22– 24^. Each cell line or cell type has a characteristic timing domain profile which can then change if the cells differentiate ^22, 25– 27^. Timing domains vary in size, ranging from a few hundred Kbp to around 10 Mbp ^22, 28, 29^. Their sizes suggest that timing domains typically comprise more than one adjacent replicon cluster ^22, 30^. The conservation of the replication timing profiles is in contrast to the considerable stochasticity in the firing of the individual origins within each timing domain.

At the cytological level, DNA replication occurs in replication *foci* within the nuclei of S phase cells. The subnuclear localisation of these replication foci changes in a predictable way during the course of S phase ^8, 15, 18, 31– 33^, reflecting progression through the replication timing programme. There are ~1,000 *foci* active at any given time in a typical somatic S phase cell ^34^, and with ~10,000 replication forks being active at any one time in S phase, each replication *foci* contains ~10 active DNA replication forks ^34, 35^. The similarity between the size and distribution of origin clusters (as observed by DNA fibre analysis) and replication *foci* (as observed by microscopy) suggests that they probably represent the same fundamental unit of DNA replication ^8, 15, 30, 36^. The DNA replicated in individual replication foci persist as foci throughout G1, S, G2, and mitosis over multiple cell cycles, suggesting that they represent a stable unit of chromosome organisation ^8, 15^.

In this paper we sought to identify genomic regions that correspond to the replicon clusters observed by DNA fibre analysis. We use a synchronisation protocol that gives a cohort of cells that enter S phase with a high degree of synchrony. At different times after release into S phase, we pulsed cells with EdU and sequenced the DNA replication ongoing during the length of time that these pulses lasted. Our high-resolution data shows the existence of discrete peaks of DNA synthesis within individual Early Timing Domains. The width of these peaks (~500 kb) is consistent with them representing individual replicon clusters, so we have named them ‘Replicon Cluster Domains’. The distribution and evolution of these Replicon Cluster Domains (RCD) suggest that their activation time varies between different cells in a population and shows a preference for a sequential ‘domino’-type activation sequence.

## Methods

### Cell culture

U2OS human osteosarcoma bone cells (U-2 OS, ATCC® HTB-96) were purchased from ATCC. Neuro-2a mouse neuroblasts cells (ATCC® CCL-131™) were also used as a control in some experiments. Cells were grown in Tissue Culture incubators (Forma Scientific CO2, water, jacketed incubator; Life Sciences Instruments), in pre-warmed (37°C) 1xDMEM (containing 4,500 mg/L glucose, 110 mg/L sodium pyruvate, 584 mg/L L-glutamine and no HEPES) (Dulbecco's Modified Eagle Medium) (Thermo Scientific/Gibco, 41966-052) medium complemented with FBS (Thermo Fisher Scientific/Gibco, 10270-106) (10% v/v/), and 100 U/ml of penicillin and streptomycin (P/S) ((Fisher Scientific/Gibco, 15140-122) (1% v/v/), at 37°C, 5% CO _2_. Cells washes were carried out with 1xDPBS (Thermo Scientific/Gibco, 14190-169) pre-warmed at 37°C.

Cells were harvested, before reaching confluency, after Trypsin-EDTA (0.05%) (Gibco, 25300-054) digest at 37°C, for 5 min; digest was stopped by the addition of 1xDMEM (pre-warmed at 37°C). Cells were spun in a centrifuge (Eppendorf centrifuge 5810) at 211xG (1.000 rpm), for 5 min at room temperature (RT), and the supernatant removed. The cell pellet was either immediately frozen in liquid nitrogen or was resuspended in a small volume of 1xDMEM and fixed in ice cold (-20°C) 70% ethanol solution overnight (or for several days) at -20°C.

### Cell cycle synchrony (G0/G1 cell cycle arrest)

Cells were first grown in pre-warmed (37°C) 1xDMEM (Dulbecco's Modified Eagle Medium) medium complemented with FBS (10% v/v/) and penicillin and streptomycin (P/S) (1% v/v/), at 37°C, 5% CO _2_. After 24 hours the medium was replaced with fresh medium 1xDMEM complemented with 0.1% (v/v/) FBS and 1% (v/v) P/S, and cells were grown for three more days. Cells washes were carried out with 1xDPBS pre-warmed at 37°C. Cells were washed with 1xDPBS prior to trypsin digest, and cell harvest was carried out as described in Cell cycle synchrony (G1/S phase cell cycle arrest).

### Cell cycle synchrony (G1/S phase cell cycle arrest)

Cells were inoculated in 1xDMEM+FBS+P/S medium and allowed to grow for 30 hours. Cells were then grown in fresh 1xDMEM+FBS+P/S medium containing Thymidine (2.5mM) (Sigma, T1895-5g) for a further 24 hours. Cells were then grown for 10 hours in 1xDMEM+FBS+P/S medium only. Finally, the cells were grown for six hours in 1xDMEM+FBS+P/S medium containing L-mimosine (0.5mM) (Sigma, M0253-100mg). All medium was pre-warmed at 37°C prior to being added to the cells.

After the L-mimosine treatment, cells were immediately washed with ice cold (4°C) 1xPBS, prior to adding fresh medium (pre-warmed at 37°C) to the cells. Cells were harvested using Trypsin-EDTA (0.05%) (5 min incubation, at 37°C), followed by enzymatic neutralisation by adding cold (4°C) 1xDMEM. Cells were immediately collected and spun in an Eppendorf centrifuge (5804R) (at 4°C) for 10 minutes, at 180 rcf. The cell pellet was either immediately frozen in liquid nitrogen or was resuspended in a small volume of 1xDMEM and fixed in ice cold (-20°C) 70% ethanol solution overnight (or for several days) at -20°C. These cell cycle synchronised cell populations are referred to, in this paper, as TM cell cycle cell populations.

### Incubation with EdU

Prior to harvesting cells were incubated with 1xDMEM+FBS+P/S medium containing 40 µM 5-ethynyl-2’-deoxyuridine (EdU) (Invitrogen, A10044) for different amounts of time, depending on the time course experiment being carried out. EdU was used from a stock of 10 mM EdU in dimethyl sulfoxide (DMSO); control cells not exposed to EdU had the same amount of DMSO added to the medium.

### Cell culture (for next generation sequencing - NGS)

Approximately one million cells were inoculated in 20 ml medium in a 150 mm diameter plate. Each biological replica, for each time point experiment, was carried out using three 150 mm diameter plates. Cell cycle synchronous cells experiments were prepared as described above. Asynchronous cells experiments were carried out as described above (in Cell culture). After digestion with Trypsin-EDTA and neutralisation with 1xDMEM (at 4°C), a small aliquot of the re-suspended cells was collected, and spun in an Eppendorf 5804R centrifuge, at 180 rcf, for 5 min (at RT) and fixed in 70% ethanol solution at -20°C; these cells were used to do cell cycle analysis by flow cytometry. The remaining re-suspended cells were centrifuged at 4°C, for 10 min, at 180 rcf; the cell pellet was immediately frozen in liquid nitrogen.

### 2D flow cytometry analysis (2D FACS)

The Click-iT reaction (Click-iT Plus EdU Alexa Fluor 647 (flow cytometry assay kit); Invitrogen, C10635) was carried out on cells fixed in 2 ml of 70% ethanol (at -20°C) for at least 12 hours, in the dark. To the 2 ml of cells fixed in 70% ethanol at -20°C O/N, 3 ml of filtered 1% BSA (in 1xPBS) were added, followed by centrifugation in an Eppendorf centrifuge (5804R) at 260xG for 5 min at 4°C. The 1% (w/v) bovine serum albumin (BSA) fraction V (Roche, 10735094001) (in 1xPBS) solution was filtered with a 0.22 µm filter (Fisher, 10400031) prior to use. After removal of the supernatant, the pellet was resuspended in approximately 100 µl of remnant supernatant, and washed one more time with 1 ml of 1% BSA (in 1xPBS) followed by a centrifugation at 260xG for 5 min at 4°C. After removal of the supernatant, 250 μl of the Click-iT reaction mix (2mM CuSO _4_ II, 0.1x reaction buffer additive, 1:200 dil. Alexa Fluor 647, in 1xPBS) (Click-iT Plus EdU Alexa Fluor 647 flow cytometry assay kit; Invitrogen, C10635) was added to the cells. The Click-iT reaction was carried out in the dark, at room temperature (RT), for 30 min. The reaction was terminated by adding 0.8 ml 1% BSA (in 1xPBS). After a centrifugation at 260xG for 5 min at 4°C, and removal of the supernatant, 0.8 ml of 1% BSA (in 1xPBS) were added to the cell pellet. Cells were centrifuged again and resuspended in 500 µl propidium iodide (50 µg/ml propidium iodide (Sigma, P4864-10ml), 50 µg/ml RNaseA (DNase-free, protease-free) (Thermo Scientific, EN0531), in 1xPBS). Cells were kept in the dark for 30 min at RT, prior to flow cytometry.

Samples were analysed on a FACS Canto (Becton Dickinson) flow cytometer, using FACSDiva version 8.01. Propidium iodide was detected using 488 nm excitation and emission was detected at 530/30 nm. AF647 fluorescence was detected using 640 nm excitation and emission detected at 660/20 nm. Data analysis was carried out using FlowJo software, version 10.6.2. Whilst FlowJo is one of the leading software packages available for analysing flow cytometry data, the data can also be analysed by free software packages such as Cytospec.

### Cell sorting

Cells were sorted on the Influx (Becton Dickinson) or on the SH800 (Sony Biosciences). Results yielded from both cell sorters did not differ significantly. In both cases, propidium iodide fluorescence was detected using 488nm excitation and fluorescence detected at 580/30nm on the Influx and 600/60nm on the SH800.

All samples were cell sorted except the asynchronous (AS) cells labelled with EdU for one hour and for 24 hours (AS1 and AS24); the entirety of the cells harvested from the cell culture plates from these samples (AS1 and AS24) was used in the production of the respective gDNA libraries. The quality of these samples was checked by 2D FACS based on propidium iodide (PI) (Sigma, P4864-10ml) fluorescence signal and Alexa Fluor 647 (Click-iT reaction) fluorescence signal. All the other cell samples that were cell sorted, were first PI stained, and RNAseA treated (Thermo Scientific; EN0531), prior to being sorted/collected.

### Genomic DNA extraction

Genomic DNA (gDNA) extraction was carried using the DNeasy Tissue & Blood kit (Qiagen, 69504), following the protocol recommended (for mammalian cells) by the manufacturer. Elution of the genomic DNA from the columns was carried out in two consecutive elution steps each with 200 µl 1xT.E. (0.1mM EDTA) pH 8.0. The integrity of the extracted gDNA was checked in an agarose (0.8% (w/v) agarose in 1xTBE) electrophoresis gel, and was quantified in a spectrophotometer (Geneflow Nanophotometer, Geneflow).

H _2_O was added to the eluted gDNA to give a final volume of 250 μl. The gDNA was sonicated in an ice-cold water bath in a Diagenode Bioruptor (high power, 15 min) with alternating 30 second cycles of sonication. The size range of gDNA fragments checked on a 2.5% agarose gel ranged from ~100–900bp, with the bulk being between ~200–600bp. Sonicated gDNA was precipitated (0.31 µg/µl glycogen, 70% ethanol (molecular biology grade), 83mM NaOAc pH 5.2), overnight at -80°C. After centrifugation (20817xG, 40 min, 4°C) the pellet was washed twice with 70% ethanol, dried and resuspended in 15 µl 10mM Tris-HCl pH 7.4.

### Click-iT reaction on sonicated gDNA

Click-iT Nascent RNA Capture kit (Life Technology/Thermo Fisher, C10365) was used to biotinylate the EdU present in the sonicated gDNA, following the manufacturer’s instructions. The 50 µl Click-iT reactions were precipitated (50 µg/ml glycogen, 0.47M NH _4_OAc, 87.5% ethanol, -80°C overnight); after centrifugation (20817xG, 40 min, 4°C) pellets were washed twice with 75% ethanol, dried and resuspended in 40 μl H _2_O. The samples were quantified in a spectrophotometer (Geneflow Nanophotometer, Geneflow).

### gDNA library construction

For each sample, three reactions of End-repair (with 1 µg of gDNA per reaction) were carried out using the Next ULTRA library prep. kit for Illumina (NEB, E7370S). This was followed by the ligation of the Next Adaptor for Illumina to the ends of the DNA fragments (NEB Next Multiplex Oligo for Illumina (index primer 1) kit (NEB, E7335L)). Finally, USER enzyme digest was carried out. The manufacturer’s protocol was followed in all these three steps. After the digest with the USER enzyme (NEB, M5505S), the DNA was precipitated (0.47M NH _4_OAc, 50 µg/ml glycogen, 87.5% ethanol, -80°C, overnight) and resuspended in H _2_O. The three reactions from each sample were bulked together. DNA was purified using a (Mini elute PCR purification kit (Qiagen, 28004) and the eluted DNA quantified in a spectrophotometer. One percent of the volume of the eluted samples was used for sequencing the full-length genome DNA of that same sample (the 1% sequence data). The remaining 99% of the samples were used for the biotin pull-down.

### Biotin-tagged gDNA library pull-down for next generation sequencing

Pull-down was carried out with all gDNA libraries except the gDNA libraries made from the G0/G1 cells. Pull-down was carried out using streptavidin coated magnetic Dynabeads (MyOne C1, Thermo Fisher/Invitrogen, 65001) and low adherence tubes (Axygen max. recovery, 1.5 ml, Corning, MCT-150-L-C). In each pull-down, 30 µl magnetic beads were used. Beads were washed three times with cold 1x B&W buffer (5mM Tris-HCl (pH 7.4), 0.5mM EDTA (pH 8.0), 1M NaCl); 60 µl cold 2x B&W buffer was added to the beads plus the biotinylated-gDNA. Binding was carried out in the dark, for >1 hour at room temperature on a tube roller (roller mixer SRT9, Stuart). Tubes were then placed in a magnetic rack (Genetics/FastGene MagnaStand 1.5, FG-SSMAG1.5) for 3 min, and all solution was removed. Beads were washed with cold 1x B&W buffer; the tube was then placed in the magnetic rack for 3 min and the supernatant was removed. After three more washes the beads were washed with H _2_O and then suspended in 25 µl H _2_O to give the pulled-down sample. For quality control of the pull-down, 1 µl of this sample was used; another 1 µl was used for qPCR to determine the appropriate indexing/amplification cycle. The remaining 23 µl of the pulled-down sample was used for the indexing of the library, using a combination of universal primer and an index primer (NEB Next Multiplex Oligo for Illumina (index primer 1; NEB, E7335L). The indexing PCR reaction pulled-down sample was carried out directly on the beads, using a 1:100 dilution of the bead’s suspension and primers for the adaptor region (NEBadqPCR_F; ACACTCTTTCCCTACACGACGC and NEBadqPCR_R; GACTGGAGTTCAGACGTGTGC) then dual-indexing was done using NEB E7600S or E7780S, aiming to obtain a total of 100–1,000 ng indexed DNA (typically 8–18 cycles of amplification).

Quality control of the pull-down, prior to the indexing of the library, was carried out in Eppendorf Mastercycler PCR machine as follows: First, the 1 µl of beads from the pulled-down sample and a small volume of the input 1% was amplified with eight PCR cycles using the Illumina library kit (NEB Q5 Hot Start PCR Master mix) and Next ULTRA library prep. kit for Illumina (NEB, E7370S) plus the Universal primer and the index primer 1 (NEB Next Multiplex Oligo for Illumina; NEB, E7335L) in 20 µl PCR mix (1 µM of Index 1 primer, 1 µM Universal primer, 0.5x NEB Q5 Hot Start buffer + enzyme), 1x cycle at 98°C for 30 sec, 8x cycles at 98°C for 10 sec, followed by 65°C for 1 min 15 sec, and a final 1x cycle at 65°C for 5 min. A second PCR amplification of 45 cycles was carried out on 4 µl of the product of the first PCR amplification without beads, using the same primers but another Taq enzyme and PCR buffer (Thermo Fisher/Invitrogen, 10342053) in 30 µl of a PCR mix (1x PCR buffer Thermo Taq, 0.25mM dNTP, 1 µM of Index 1 primer, 1 µM Universal primer, 3 units Thermo Taq). This second PCR reaction had the following parameters: 1x cycle at 95°C for 2 min, 45x cycles at 95°C for 20 sec, 65°C for 30 sec, 68°C for 1 min 15 sec, and a final 1x cycle at 68°C for 5 min. The purpose of this second PCR was to determine whether the PCR products (run in a 2.5% agarose gel in 1xTAE buffer) matched the gDNA smear observed when the gDNA library itself was run on a 2.5% agarose gel. In the third PCR, 0.5 µl of the products of the first PCR amplification (minus beads) was used with h_intGenMit-F ( ^5’^CCTAGGAATCACCTCCCATTCC ^3’^) and h_intGenMit-R ( ^5’^GTGTTTAAGGGGTTGGCTAGGG ^3‘^) primers ^37^, as well as Taq enzyme and PCR buffer (Thermo Fisher/Invitrogen, 10342053) in 20 µl PCR mix (1x PCR buffer Thermo Taq, 0.25mM dNTP, 0.8 µM of h_IntGenMit-F primer, 0.8 µM h_IntGenMit-R primer, 1 unit Thermo Taq). The cycling parameters of the third PCR reaction were: 1x cycle at 95°C for 2 min, 33x cycles at 95°C for 45 sec, 60°C for 1 min, 68°C for 2 min, and a final 1x cycle at 68°C for 5 min. The purpose of this third PCR was to assess if the pull-down samples contained pulled-down DNA from the U2OS cells.

### Sequencing data processing

Quality control of FASTQ reads was carried out with *FastQC* ver. 0.11.4 and revealed significant contamination with adapter sequencies. Subsequently, the adapters were trimmed using *TrimGalore* ver. 0.5.0. Reads were mapped to the human reference genome GRCh38, release 94, obtained from Ensembl, using *Bowtie2* ver. 2.3.0 ^38^. Resulting BAM files were filtered for read quality (MAPQ > 10), sorted and indexed with *Samtools* ver. 1.9 ^39^. BAM files were converted into BED files, which were binned into bedGraph format in 10,000- and 50,000-bp bins, using *Bamtools* ver. 2.27.1 ^40^. The scripts created for this analysis can be found in the Github repository:

https://github.com/bartongroup ^41^

### Background subtraction and normalisation

bedGraph files contain distribution of reads (genomic tracks) for both pull-down ( *P*) and genomic input control ( *C*). Background subtraction and normalisation is based on the CISGenome normalization ^42, 43^. For a given pull-down and control the score is defined as reads per million, *P _i_* and *C _i_*, respectively, where *i* indicates position in the genome (across all chromosomes). We assume that the pull-down consists in part of the genomic background, *B _i_*, since pull-down procedure is never 100% effective and the real pull-down signal: *P _i_* = *B _i_* + *S _i_*. We assume that the background present in the pull-down has the same distribution across chromosomes as the control, with an unknown normalisation factor, *B _i_* = *rC _i_*. The purpose of normalisation and background subtraction is to find *r*.

A plot of log ( *P _i_*/ *C _i_*) versus log ( *P _i_* + *C _i_*) can be approximated by a broken line with two segments. The horizontal segment, at low counts, represents genomic regions were pull-down and background are equal, that is with no signal, *S _i_* = 0. A change in total count, *P _i_* + *C _i_* ≈ (1 + *r*) *C _i_* does not affect a constant ratio of *P _i_*/ *C _i_* ≈ *r*. In contrast, the part of the plot with a positive slope represents regions with positive signal *S _i_* > 0 where *P _i_*/ *C _i_* = *r* + *S _i_*/ *C _i_*. Thus, *P _i_*/ *C _i_* ∝ *S _i_* where signal is strong, *S _i_* ≫ *B _i_*. By fitting these data with a broken line, a break point *b* can be found. All data with log ( *P _i_* + *C _i_*) < *b* belong to genomic regions with no signal. This set is denoted as *G* = { *i*: log( *P _i_* + *C _i_*) < *b*}. The CISGenome method finds the normalisation factor as


$${\hat{r}}_{cis}=\frac{\sum_{i\in G}P_{i}}{\sum_{i\in G}C_{i}}.$$

This approach, however, does not work well with data with high coverage of peaks, as the background-isolation method is not perfect and *G* contains, in part, regions with some signal. The distribution of *P _j_*/ *C _j_*, where *j* ∈ *G*, is not symmetric and contains a high-count tail. Here, the peak of the *P _j_*/ *C _j_* distribution (the mode) is chosen to estimate the normalisation factor $\hat{r}$ , as it represents the most frequently found *P _j_*/ *C _j_* ratio. After this, the background-subtracted signal is found as *S _i_* = *P _i_* – $\hat{r}$ *C _i_*, for each bin *i*.

### Data analysis and deposition

Preliminary data investigation and analysis, normalisation and background subtraction were done in R. The code is available at GitHub ( https://github.com/bartongroup) and archived with DOI 10.5281/zenodo.7639072 ^41^. The 10–40, 40–70, 70–100 and 100–130-minute data used in most of the paper came from a single experiment that was processed in parallel, but the 0–40 min data came from a separate experiment. All datasets are provided at Biostudies (S-BSST966). The replication timing data for U2OS was from https://data.4dnucleome.org/, accession numbers: 4DNES99LXRYK and 4DNES1P18J2X.

Further analysis was done in the Swift programming language (Swift 5.7.2) using the Xcode development environment (Xcode version 13.3). The analysis code is available together with the R code at GitHub ^41^. It consists of: i) a DataCentre class which contains all the core analysis functions; ii) an EarlyRepDataSet class which stores the early replication signal and derived information from it, including the wavelet analysis signals; iii) a U2OSTimingDomains class which stores the information from the previously published replication timing analysis on U2OS cells; iv) a WaveletAnalysis file which contains the functions for performing wavelet analysis with the Ricker wave; v) an adjacentValueSimilarityMetric function that returns a metric indicating the similarity between adjacent values in an input array; vi) a Gaussian struct which supports the Gaussian function; vii) a flatToppedGaussian struct that supports a Gaussian function with a flat middle section; viii) a NelderMead class that performs parameter fitting using the Nelder-Mead algorithm; ix) an AppDelegate class which provides Controller function to mediate between the graphical user interface and the analysis classes; and x) View structures and classes that provide the graphical user interface (MainMenu, JBGraph, Decimal+Normalisation and JBGraphSheets). The operation of most of the functions are described in the main text and figure legends.

The adjacentValueSimilarityMetric function works as follows. Given an input array of n values, the function returns a metric which indicates the similarity between adjacent values in the input array. These values are based on the n-1 absolute differences between adjacent values in the array. First, the function calculates the mean value of the absolute differences between adjacent values in the array (AdjacencyDifferenceForArray; ADFA). The function then calculates the mean value of the absolute differences between adjacent values in a sorted version of the array (AdjacencyDifferenceForSortedValues; ADFSV); this is equivalent to the absolute difference between the largest and smallest values divided by the number of items in the array. Next, the function calculates the mean value of the absolute differences between adjacent values in randomly-sorted versions of the array (MeanAdjacencyDifferenceForAllPermutations; MADFAP); this is equivalent to the average difference between all non-identical pairwise comparisons of the values in the array. The AdjacencyDifferenceForArray metric is then normalised so that a value of one means that the adjacent values are ordered in a maximally similar way; a value of zero means random similarity; and negative values indicate anti-similarity. This is calculated for the return value as:


$$\frac{1-(ADFA-ADFSV)}{MADFAP-ADFSV}$$

## Results

### Creating a highly synchronous S phase population

**Figure 1.**
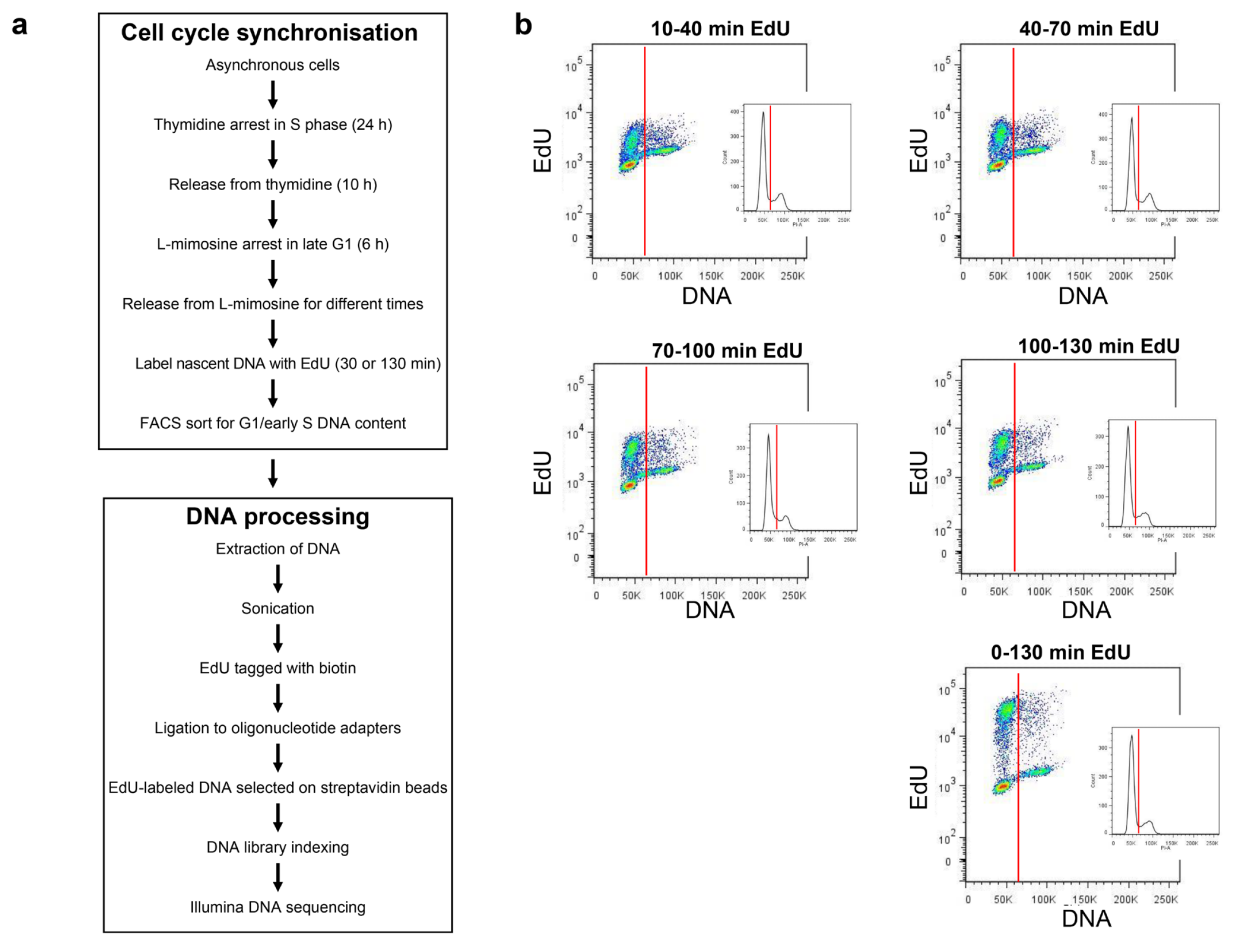
Synchronisation of early S phase cells. **a**) Upper panel: description of the procedure for synchronising U2OS cells in early S phase; lower panel: description of method for isolating replicating DNA from synchronised cells. **b**) Flow cytometry of synchronised cells. Prior to analysis cell cultures were supplemented with EdU for either 30 or 130 mins at the indicated times after release from L-mimosine. EdU incorporated into DNA was labelled with Alexa Fluor 647 and total DNA was stained with Propidium Iodide. Cells were then analysed by flow cytometry: the x-axis shows DNA content (propidium iodide) and the y-axis EdU content. The red vertical lines indicate the cut-off used in preparative cell sorting experiments to include only cells with a near-G1 DNA content.

In order to label DNA sequences that represent individual replicon clusters, we devised a protocol by which cells enter S phase with a high degree of synchrony. U2OS cells were synchronised using one round of thymidine treatment followed by release into normal medium and then treatment with L-mimosine ^14, 44– 46^ ( Figure 1a). Release from L-mimosine allowed cells to enter S phase with a degree of synchrony significantly higher than we could obtain by release from thymidine arrest. Because L-mimosine is a hypoxia mimetic and can induce double-strand breaks ^47– 50^, the protocol was designed to minimise the amount of time that cells were exposed to L-mimosine ( Figure 1a, top panel). At different times after L-mimosine release, newly replicated DNA was labelled with 30-minute pulses of the thymidine analogue 5-ethynyl-2’-deoxyuridine (EdU); the DNA was sonicated into fragments ranging from 100–900 bp, the EdU derivatised with biotin, captured on magnetic beads and subjected to DNA sequencing ( Figure 1a, bottom panel). DNA reads were then mapped back onto the reference human genome and read count was collected in 10 or 50 kb bins.

The EdU in small aliquots of cells from the different treatments was labelled with Alexa Fluor 647 for flow cytometry. Figure 1b shows 2-dimensional flow cytometry profiles showing DNA content (propidium iodide) versus EdU incorporation (Alexa Fluor 647). It took ~10 minutes after L-mimosine release for the first cells to start incorporating EdU and new cells continued to enter S phase for ~45 minutes (Underlying data: Supplementary Figure S1) ^51^. Some cells remained in G1, possibly because they had not completed progression through G1 or because they had acquired double strand breaks caused by the thymidine + L-mimosine treatment. Aliquots of cells were pulsed with EdU at 10–40, 40–70, 70–100 and 100–130 minutes after L-mimosine release. Replicating cells continued to incorporate EdU at a constant rate and could be judged to be moving through S phase by an increase in total DNA content. This increase in DNA content can be seen in synchronised cells pulsed continuously with EdU for 130 min ( Figure 1b, 0–130 min). Despite the synchrony protocol, some cells at different stages of S phase were still present in the whole cell population. Hence, to yield only cells labelled with EdU at the early stages of S phase we used FACS to select cells with a near-G1 DNA content (vertical red line in Figure 1b).

We examined different methods for normalising the results from DNA sequencing. To control for amplification and mappability biases, we sequenced the entire DNA content from synchronous and asynchronous cells plus or minus EdU labelling and streptavidin pull-down, as well as non-pulled-down samples. Reads from the experimental samples were normalised to the total number of counts in millions. Then, genomic background was subtracted using an approach based on CISGenome normalization ^42, 43^ (see Methods). We tested a number of possible controls for normalisation (Underlying data: Supplementary Figure S2) ^51^ and decided to use for normalisation the DNA from each sample prior to pull down, hence avoiding any potential sample-to-sample variation. 

**Figure 2.**
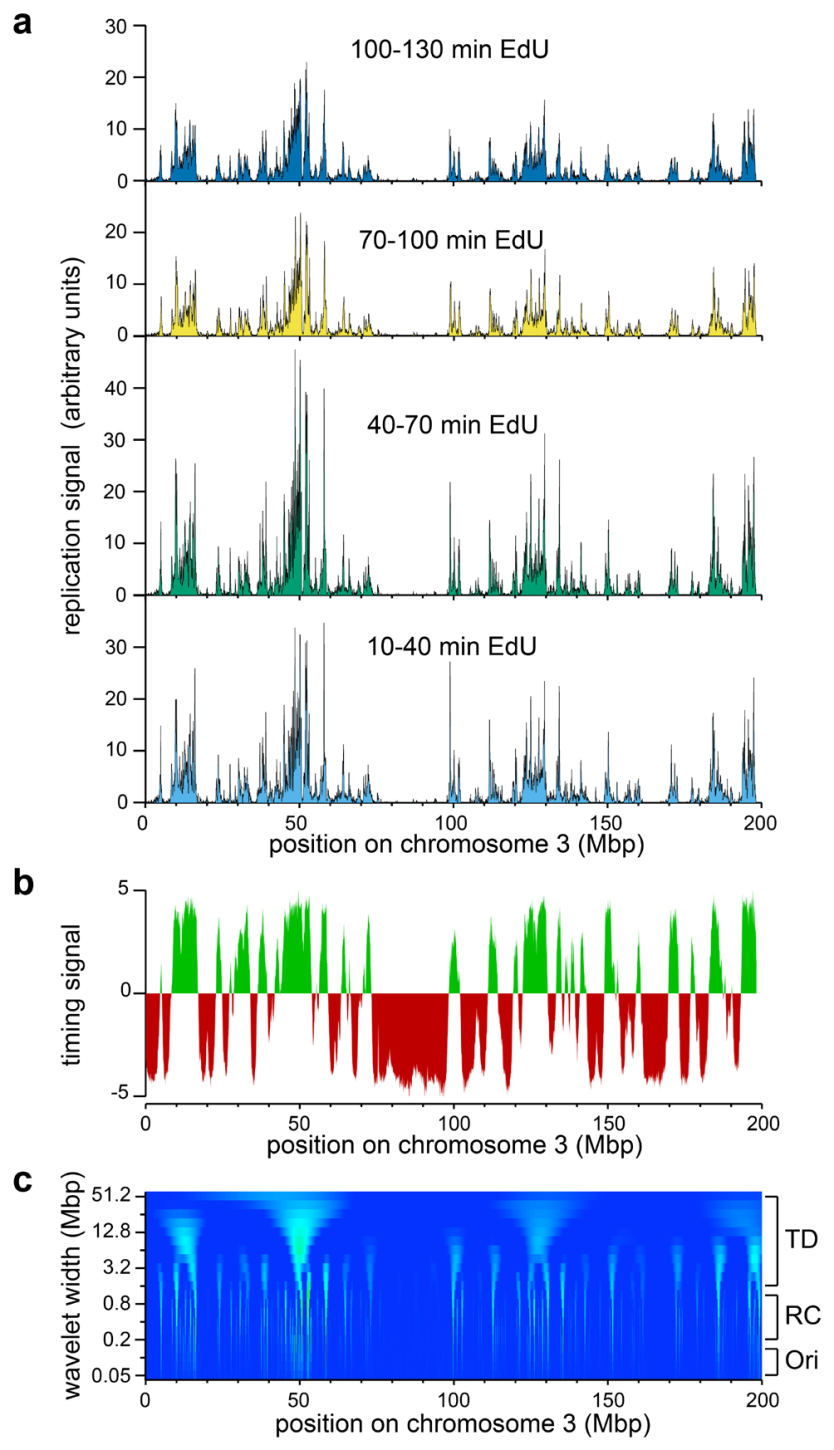
Early replication signals on chromosome 3. U2OS cells were synchronised in early S phase with thymidine and L-mimosine as described in Figure 1a, and were pulsed for 30 mins with EdU at different times after L-mimosine release. EdU labelled DNA was isolated and sequenced as described in Figure 1a and mapped back to the genome. EdU signals were then normalised to the respective sample’s internal control reference. **a**) The normalised EdU signals on chromosome 3 are shown for the four time points. **b**) The replication timing signal for chromosome 3 from https://www2.replicationdomain.com/database.php. Early replicating DNA is shown in green and late replicating DNA in red. **c**) Heatmap (positive values only) of a wavelet analysis of chromosome 3 using a Ricker wavelet of peak width from 50 kb to 51.2 Mbp, with widths increasing by a factor of √2 between each analysis (log scale) on the y axis. The approximate size of individual replicons (Ori), replicon clusters (RC) and timing domains (TD) are indicated to the right.

The results for chromosome 3 using this protocol are shown in Figure 2a, and data for all chromosomes is shown in Underlying data: Supplementary Figure S3 ^51^. Several things are apparent from this data. The replication profiles are complex, with peaks at a range of different sizes. The profiles are also remarkably consistent across the four different time points, though sharp peaks at the earlier times tend to spread out at later times.

We first asked how our early replication data conformed to published data on replication timing domains. Figure 2b shows the early (green) and late (red) replication timing domains for U2OS cells. There is remarkable concordance between the timing domain results and our early replication results. Genome-wide, 94% of the 10–40 min early replication signal falls into early timing domains. This is consistent with recent data on single DNA fibres which shows the high degree of stochasticity that occurs in origin firing ^48^. However, the early replication peaks show much more fine structure, consistent with the idea that they represent the earliest active replicon clusters in the early timing domains.

The early replication signal can be interpreted at three different scales: the scale of individual replicons (~100 kb), replicon clusters (~500 kb) and timing domains (multi-Mbp scale). Wavelet analysis provides a means of analysing signals like this at different scales. Wavelet analysis of the 10–40 min signal is shown in Figure 2c. A wavelet (in our case the Ricker wavelet, Underlying data: Supplementary Figure S4) ^51^ of a particular width is moved across the early replication data and the two signals convolved to show how much the mother wavelet is matched at different places in the replication signal; this process is then repeated with wavelets of different widths. Figure 2c shows the results when we performed this process with Ricker peak widths from 50 kb ( Figure 2c bottom) to 51.2 Mbp ( Figure 2c top). Features potentially corresponding to timing domains (TD), replicon clusters (RC) and individual replication origins (Ori) are visible in the heatmap.

In the rest of this paper, we first consider the potential replicon clusters in the 10–40 min time point (EdU pulse from 10–40 mins) and then consider how these replicon clusters develop over time.

### Replicon cluster domains

**Figure 3.**
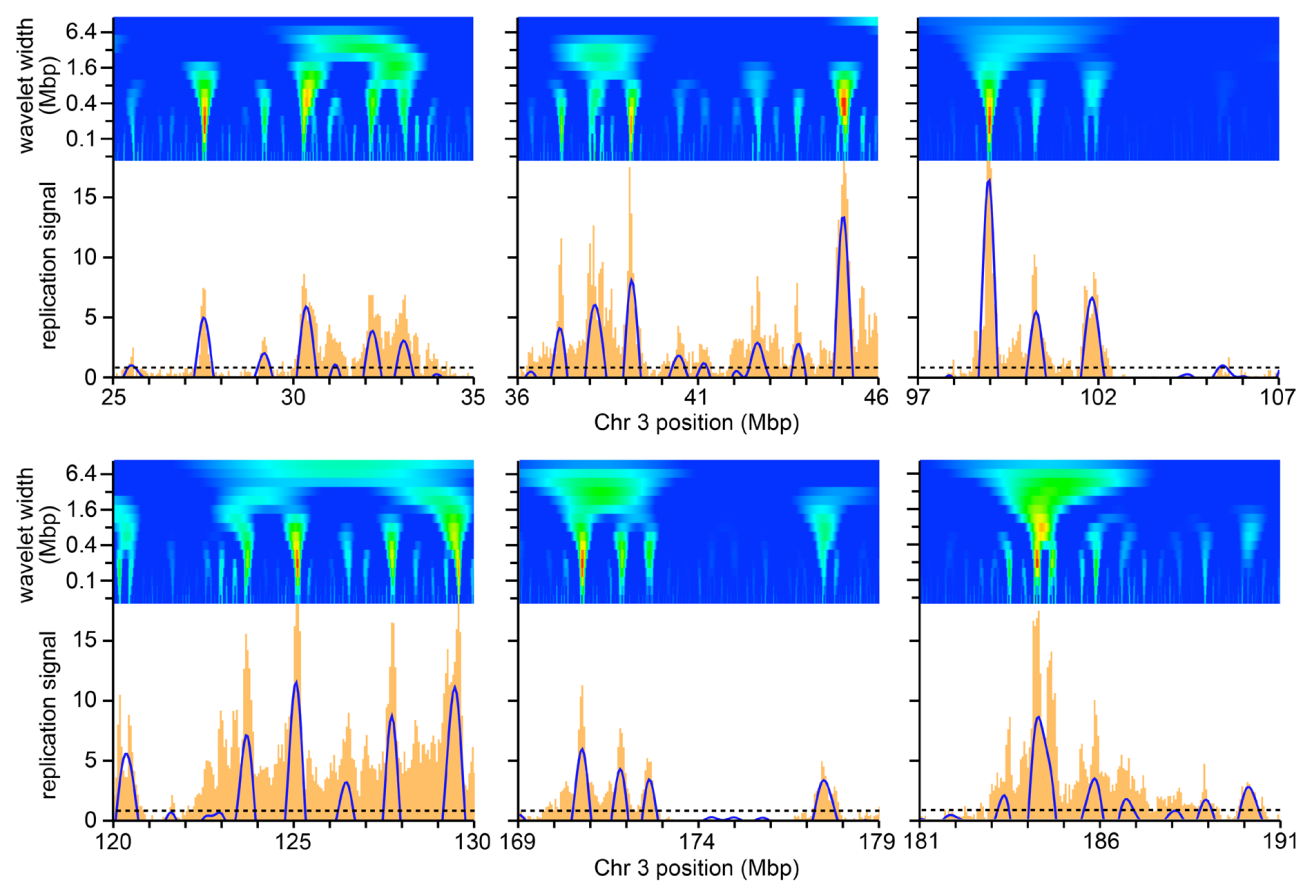
Illustrative results of selected regions on chromosome 3. Orange bars show the early replication signal from the first time point (EdU labelling from 10–40 mins) on six selected 10 Mbp regions of chromosome 3. Blue lines show the wavelet analysis results with a wavelet peak widths of 500 kb (positive values only). The horizontal dashed line shows the cut-off for calling a wavelet peak as set at the 70 ^th^ percentile for peaks in late replicating domains (see main text for rationale). Above each replication signal is heatmap of wavelet analysis using a Ricker wavelet of peak widths ranging from 50 kb to 9 Mbp with widths increasing by a factor of √2 between each analysis (log scale) on the y axis and using data in 10 kb bins to allow analysis with the smallest wavelets.

We used wavelet analysis in order to show that the most prominent component of the early replication signal is at ~500kb in size, consistent with the expected size of replicon clusters ^8, 15^. To provide a detailed example of early replication, Figure 3 shows the 10–40 min signal for six 10 Mbp regions of chromosome 3 (orange bars, mapped onto 50 kb bins). Any two forks initiated from a single origin could travel 60–120 kb (2 x 30 mins x 1-2 kb/min) if they were in the first cells that entered S phase in this sample, and so could represent the finest features observed here (width of one or two orange bars). Most replicon clusters would be expected in the 300–800 kb range, and so could represent the broader peaks observable at this scale.

**Figure 4.**
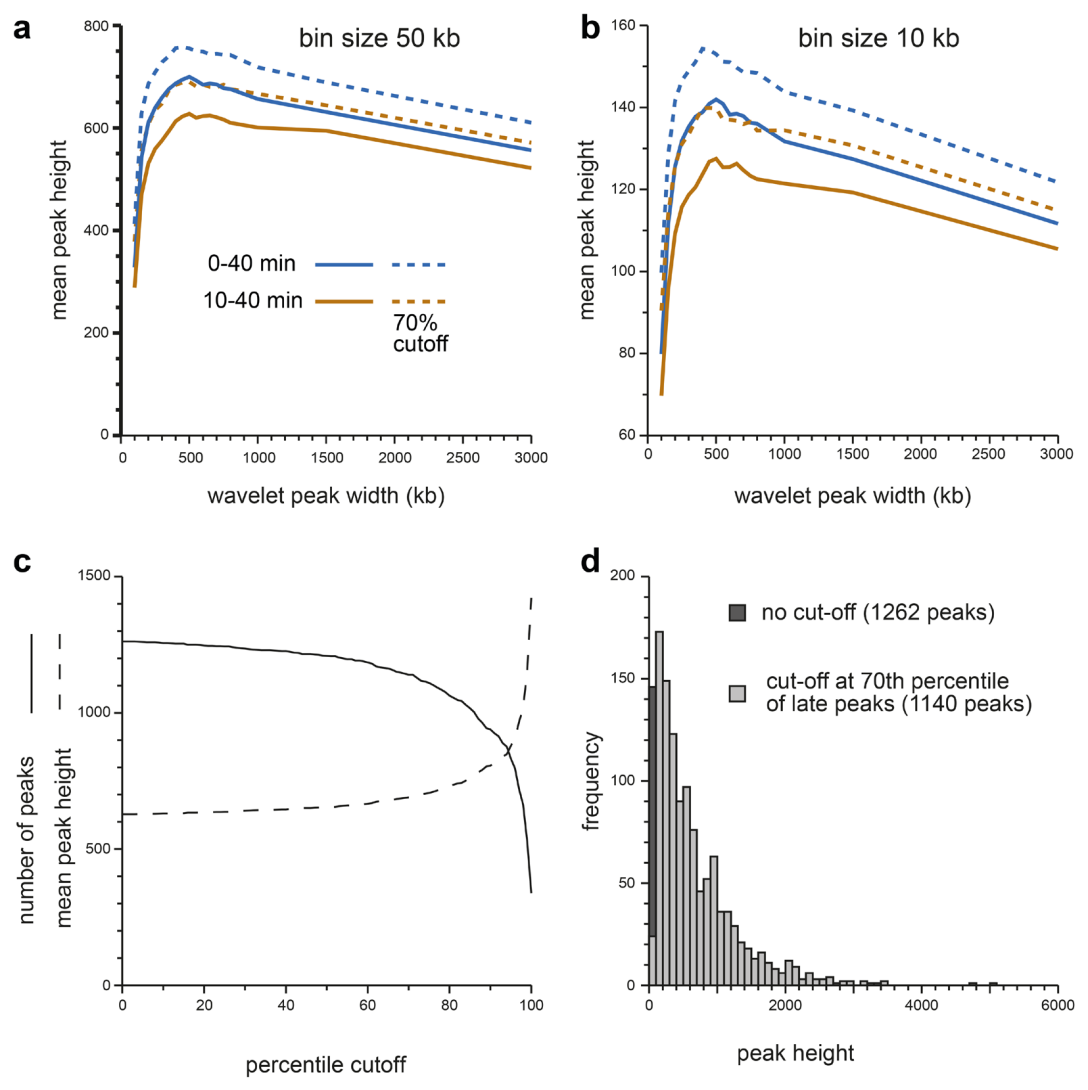
Metrics from a genome-wide wavelet analysis. **a**, **b**) The early replication signals from the first time point (EdU labelling from 10–40 mins, brown lines) or an extended first time point (EdU labelling from 0–40 mins, blue lines) with reads mapped onto 50 kb bins (panel **a**) or 10 kb bins (panel **b**) were clipped to include only the early timing domains and were analysed using a range of closely-spaced wavelets (peak widths of 100, 150, 200, 250, 300, 350, 400, 450, 500, 550, 600, 650, 700, 750, 800, 1000, 1500 and 3000 kb). Peaks were then called optionally using a minimum peak hight representing the 70 ^th^ percentile for peaks in late replicating domains (solid lines without cutoff, dashed lines with cutoff). The height of each peak was recorded and the total genome-wide sum for each data point is plotted. **c**) The early replication signal from the first time point (EdU labelling from 10–40 mins) was clipped to include only the late timing domains and analysed using a wavelet with 500 kb peak width. The peak heights were sorted in order and expressed as a percentile. The early replication signal from the first time point (EdU labelling from 10–40 mins) was then clipped to include only the early timing domains and analysed using a wavelet with 500 kb peak width. Peak calling was then performed using as a cut-off the wavelet peak height at different heights derived from the late-replicating DNA. Solid line shows the number of wavelet peaks (solid line) and the mean wavelet peak height (dashed line) called in the early timing domains using different percentile cut-offs derived from the late timing domains. **d**) The distribution of wavelet peak heights obtained from applying a wavelet of 500 kb to the first time point (EdU labelling from 10–40 mins) clipped to include only the early timing domains. The darker bar shows the effect of using a 70 ^th^ percentile cutoff derived from the replication signal in late timing domains, which removes 87 of the smallest wavelet peaks.

Figure 3 also shows heatmaps of wavelet peak width ranging from 50 kb to 9 Mbp (using data in 10 kb bins to allow analysis with the smallest wavelets). It is evident from these examples that the strongest wavelet results are with peak widths in the region of 200–800 kb, in the expected size range of replicon clusters. To determine whether this represents a global feature of early replicating DNA, we separated DNA early timing domains from later replicating DNA using the timing domain data (the green portions in Figure 2b and Underlying data: Supplementary Figure S3) ^51^ and then determined the mean height of all wavelet peaks in the early replication domains. Figure 4a shows this analysis for replication data in 50 kb bins and Figure 4b shows the data in 10 kb bins. We also compared DNA labelled from 10–40 mins (brown lines) with DNA labelled from 0–40 mins (blue lines). The results were similar in all analyses: the mean peak height rose rapidly from 50 kb wavelet widths to 500 kb wavelet widths, and then fell slowly. This is consistent with the visual analysis of the heatmaps ( Figure 2c and Figure 3) and suggests that the most prominent features in all the early replicating regions have a width of ~500 kb, as expected of replicon clusters. On this basis we decided to use a wavelet width of 500 kb to identify these features which we call ‘Replicon Cluster Domains’.

The blue arcs in Figure 3 show the Replicon Cluster Domains identified with a 500 kb wavelet peak width. In order to further analyse the behaviour of these Replicon Cluster Domains, we decided to set a minimum height for calling them. We started by examining the peaks identified in the late timing domains using a wavelet width of 500 kb (the red portions in Figure 2b and Underlying data: Supplementary Figure S3) ^51^ as this largely consists of features that look like background levels of EdU incorporation. We explored the effect of setting the cut-off for peak recognition at different percentiles of the peak heights in the late timing domains. Figure 4c shows the effect on Replicon Cluster Domain identification in early timing domains of different percentile cut-offs. There were a total of 1262 wavelet peaks in early timing domains (wavelet width 500 kb). Setting a minimum cut-off at the 70 ^th^ percentile of peaks in late replicating domains (i.e., removing 70% of the peaks in late domains) removed 122 (9.7%) of the smallest peaks in the early timing domains, leaving 1140 Replicon Cluster Domains. Cut-off percentiles >70% started to significantly reduce the number of Replicon Cluster Domains in the early domains. Figure 4d shows the distribution of wavelet peak heights in the early timing domains and the effect of implementing the 70 ^th^ percentile cut-off. Establishing this 70 ^th^ percentile cut-off did not significantly change the 500 kb optimum wavelet peak width (dashed lines in Figures 4a and 4b). The 70 ^th^ percentile cut-off value is shown as a dashed horizontal line in Figure 3.

**Figure 5.**
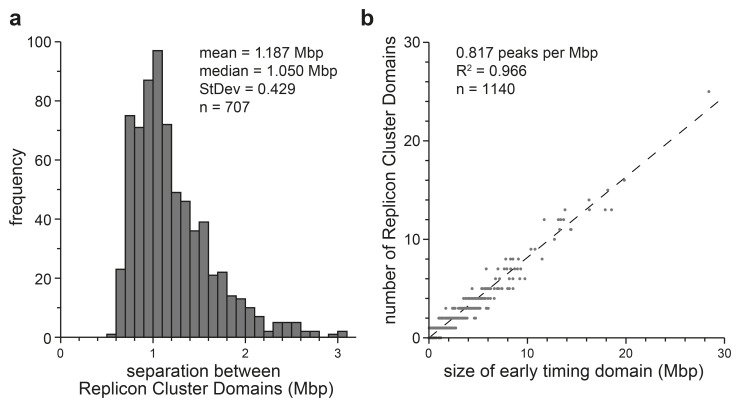
Separation between Replicon Cluster Domains. A wavelet of width 500 kb was applied to the first time point (EdU labelling from 10–40 mins) clipped to include only the early timing domains (data mapped in 50 kb bins). Wavelet peaks falling below the 70 ^th^ percentile of peaks identified in late timing domains were removed. The remainder of the peaks were classified as Replicon Cluster Domains. **a**) The distance between adjacent Replicon Cluster Domains identified within each early replicating domain was recorded. The frequency distribution of the separation between adjacent peaks is shown. **b**) The number of Replicon Cluster Domains identified in each early timing domain is plotted against the size of the early timing domain.

Figure 5 shows some metrics of the 1140 Replicon Cluster Domains identified in this manner. Figure 5a shows that the separation between Replicon Cluster Domains is fairly uniform, with a mean of 1.187 ± 0.43 Mbp. Since the width of each Replicon Cluster Domain is about 0.5 Mbp, this means that Replicon Cluster Domains peaks are quite closely packed together in early timing domains. Figure 5b shows that there is a clear proportionality between the size of a timing domain (x axis) and number of Replicon Cluster Domains it contains (y axis). This means that there is a fairly close packing of Replicon Cluster Domains within all early timing domains.

The >20-fold variation in the height of the replication peaks representing the Replicon Cluster Domains ( Figure 4d) is striking. Because our sequencing results were carefully normalised to total genomic DNA (Underlying data: Supplementary Figure S2) ^51^ higher peaks indicate more DNA replication (EdU) in that region. The difference in peak heights could be caused by a combination of two different effects: it could represent different densities of active origins in each Replicon Cluster Domain and it could represent some stochasticity in the time that Replicon Cluster Domains become active. From the extensive data on replicon sizes obtained by DNA fibre analysis it seems highly unlikely that variability in the density of replication origins could account for all of the peak height variability. Since DNA fibre analysis also suggests that the vast majority of origins fire near-synchronously in clusters, we conclude that the variability in peak height implies that there is considerable cell-to-cell variation in the time that Replicon Cluster Domains become active in different cells. We will explore this idea later.

### Growth of individual RCDs during S phase

**Figure 6.**
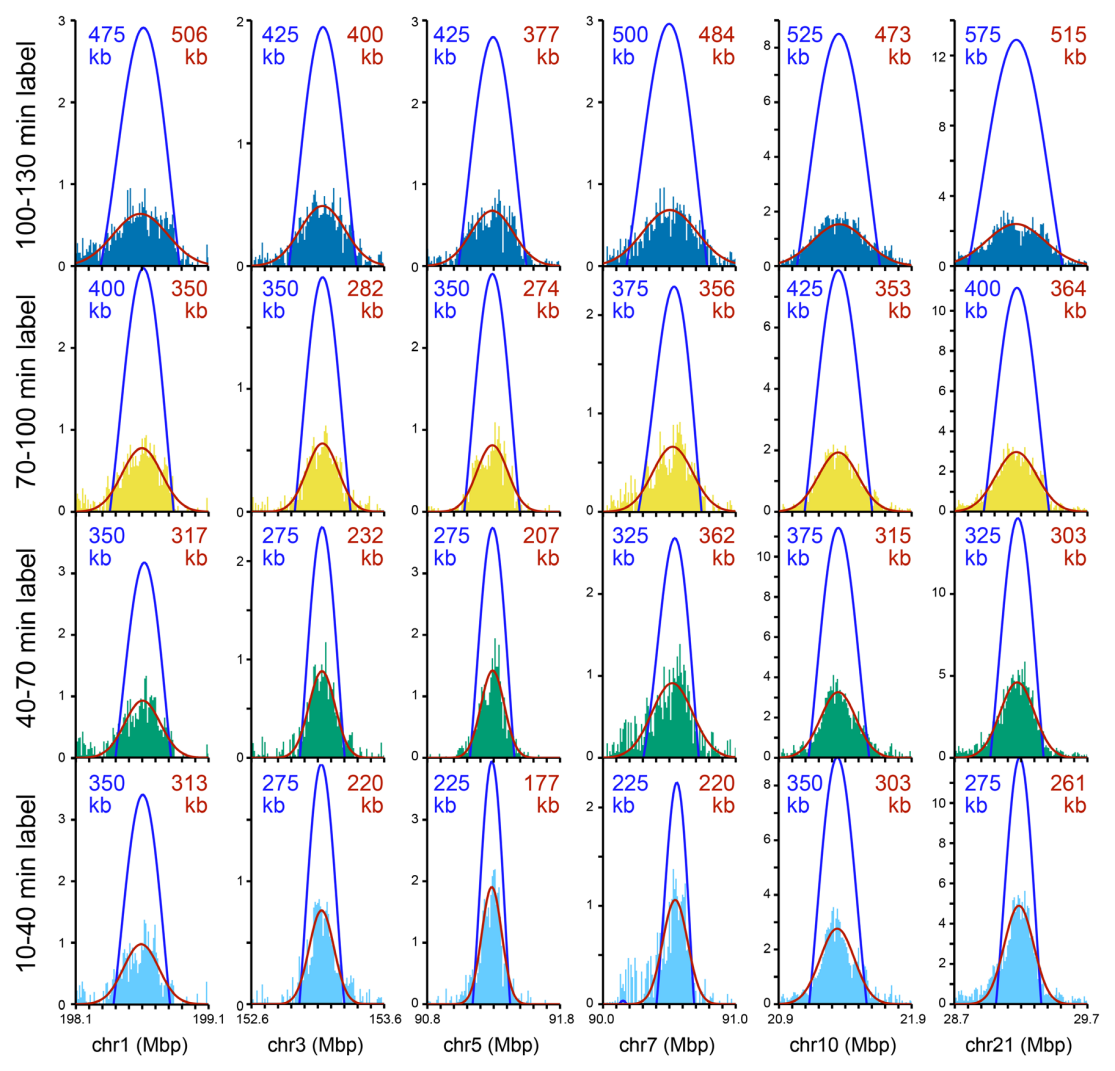
Exemplar isolated Replicon Cluster Domains. Six Replicon Cluster Domains that were substantially isolated from other replication signals were chosen as exemplars. Their replication signal in the four time points is shown (light blue, green, yellow and dark blue bars). Each Replicon Cluster Domain was analysed by a series of finely separated wavelets (in 25 kb intervals from 200 kb to 1,200 kb; data in 10 kb bins). The optimal wavelet selected is shown in blue and its width shown in blue text. The same six peaks were fitted to a Gaussian curve which is plotted in red; the red text shows the full width at half maximum of the curve.

We next turned our attention to understanding how individual Replicon Cluster Domains develop over time. The movement of the two forks at the outer edges of each replicon cluster will expand the margins of each Domain at successive time points. Visual inspection of the replication data ( Figure 2a and Underlying data: Supplementary Figure S3) ^51^ suggests that in passing through the four different time points, most peaks stay in the same position but get wider. In order to investigate this systematically and quantitatively, we used a simple algorithm to identify individual peaks that are sufficiently separated from their neighbours that a measurement of their width can be made. To do this, we started with the 1140 Replicon Cluster Domains identified as in Figure 5, using the 10–40 minute EdU data analysed by a wavelet peak width of 500 kb and implementing a 70 ^th^ percentile cut-off relative to peaks in late timing domains. We then removed any Replicon Cluster Domains where the total amount of replication signal in the 500 kb on either side of the wavelet peak was more than 25% of the replication signal under the wavelet peak. This gave 123 ‘isolated’ Replicon Cluster Domains in the 10–40 minute EdU data (listed in Underlying data: Supplementary Figure S5) ^51^. Figure 6 shows examples of six of these isolated Replicon Cluster Domains as they develop over the four time points. A broadening and flattening of the peaks over time is evident in these examples.

**Figure 7.**
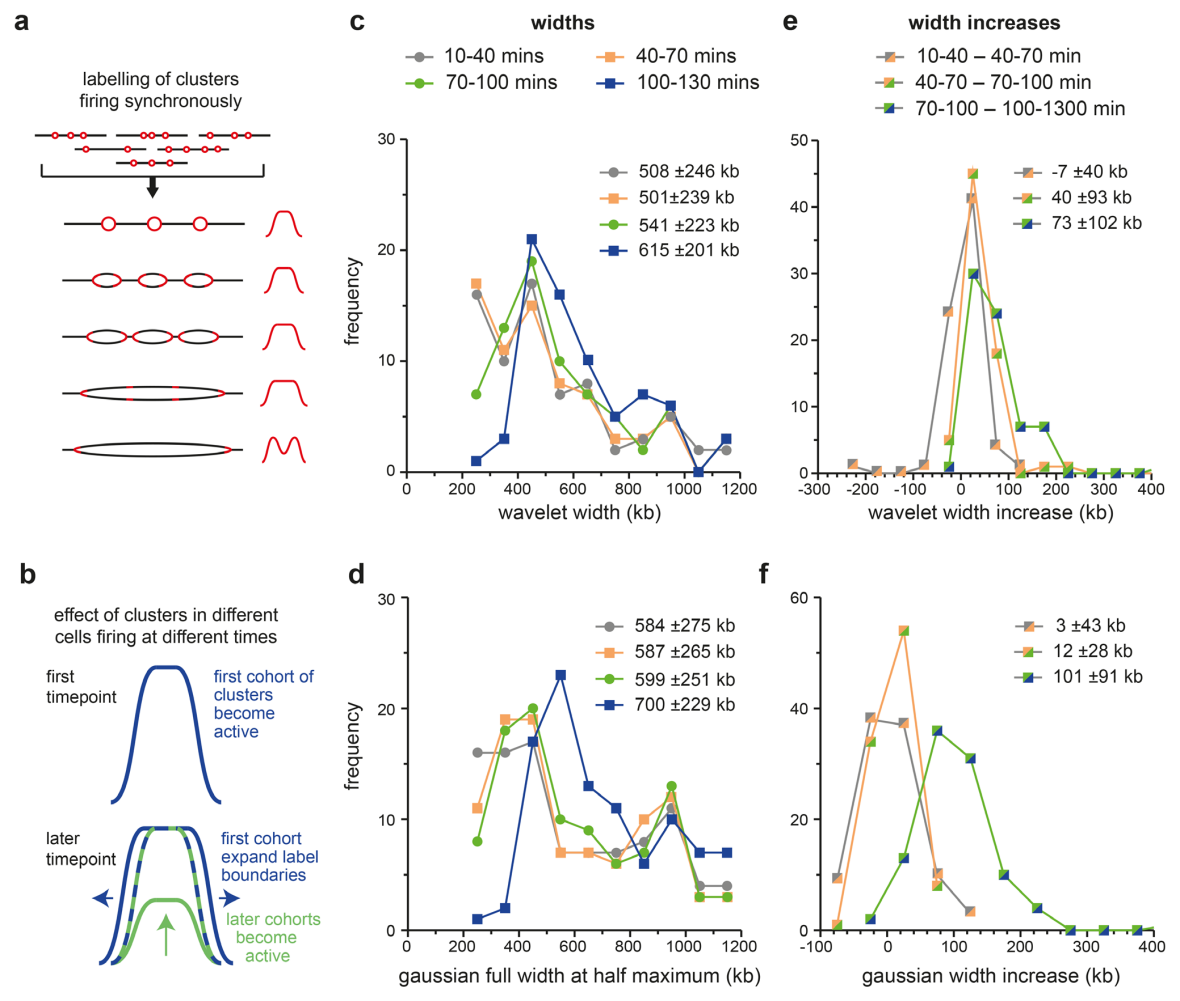
Development of isolated Replicon Cluster Domains. **a**) Schematic of an idealised Replicon Cluster Domain containing three active origins. Because of stochasticity in the selection of active origins plus near synchronous initiation within the cluster, EdU incorporation would tend to have a flat top and curve down at the edges. The width of the labelling will expand driven by the outer forks until all internal forks have terminated and incorporation is restricted to the two outer forks. **b**) Schematic of the EdU signal in a single Replicon Cluster Domain in the population of synchronised cells. Whilst in individual cells the Replicon Cluster Domain will expand its edges (blue line) the activation of the Replicon Cluster Domain in cells newly entering S phase (green line) will give an EdU signal intermediate between the two (blue-green dashed line). **c**- **f**) The Replicon Cluster Domains from the 10–40 minute EdU data as defined in Figure 5 were filtered to exclude those where the total amount of replication signal in the 500 kb on either side of the wavelet peak was >25% of the replication signal under the wavelet peak. These 123 ‘isolated’ peaks were fitted to an optimal wavelet (using a range of wavelets with peak widths at 25 kb intervals from 200 kb to 1,200 kb; panels **c** and **e**) or Gaussian curve (panels **d** and **f**) over the four time points. Peaks that fitted to the extreme values of 200 kb or 1,200 kb at any of the four timepoints were rejected. The optimal wavelet width (panel **c**) or the Gaussian width (panel **e**) are plotted. The width change between successive time points is plotted in panel **d** (wavelet) and panel **f** (Gaussian).

Figure 7a and 7b shows how idealised replicon clusters might look if they were pulsed with EdU over five successive time points. Because different origins may be selected to fire in different cells, the population will have curved (Gaussian-looking) edges, but because origin density within clusters is somewhat uniform, the curve will have a flatter top than a Gaussian curve. Origins within a single cluster fire near-synchronously, so that the flat-topped nature of the peak will increase with time. Once all internal forks have terminated, EdU incorporation will occur only at the outer edges of the cluster. The peak shapes in Figure 6 conform to some degree with the model in Figure 7a, though there is little evidence for the termination-driven peak-splitting, which suggests that most Replicon Cluster Domains are still replicating internally even at the last time point.

We analysed all 123 isolated domains using a range of wavelets with peak widths at 25 kb intervals from 200 kb to 1,200 kb and determined the best fit to the experimental data. We rejected from further analysis 51 peaks that fitted to the extreme values of 200 kb or 1,200 kb at any of the four timepoints. The location and optimal wavelet width of the remaining 72 isolated peaks is given in Underlying data: Supplementary Figure S5 ^51^ and they are shown in blue for the exemplar peaks in Figure 6.

As an alternative way of measuring the widths of isolated peaks, we took the same 123 isolated domains and fitted Gaussian curves to them using a Nelder-Mead algorithm. We rejected from further analysis 26 peaks that fitted to the extreme values of 200 kb or 1,200 kb at any of the four timepoints. The location and full width of maximum height of the remaining 97 isolated peaks is given in Underlying data: Supplementary Figure S5 ^51^ and they are shown in red for the exemplar peaks in Figure 6.

Figure 7c shows the width of the fitted wavelets, which increase in size from an average of 508 kb in the 10–40 min time point to 615 kb in the 100–130 kb time point. Figure 7d shows similar data for the width of the fitted Gaussian peaks, which increase from an average of 584 kb to an average of 700 kb. Figures 7e and 7f show how the width of each isolated peak increases in size between successive time points as analysed by wavelet fitting (7e) or Gaussian curve fitting (7f). There is no significant increase in average wavelet width between the first two time points, but between the second and third time point widths increase by an average of 40 kb (wavelet) or 12 kb (Gaussian) and between the third and fourth time points widths increase by an average of 73 kb (wavelet) or 101 kb (Gaussian).

Replication forks in early S phase U2OS cells move at 1–1.5 kb/min ^9^, so the two flanking forks in a replicon cluster would be expected to expand the width of the peak by 60–90 kb in successive labelling periods. This is in line with the two later expansion rates. The lack of significant growth between the first two time points (between the 10–40 mins and the 40–70 mins timepoints) can be explained by cells continuing to enter S phase over ~45 minutes after L-mimosine release ( Figure 1) which will tend to minimise the mean width of the labelled peak, as portrayed in Figure 7b.

A typical replicon within a replicon cluster might remain active for 37–75 min ^4, 8– 11^. Visual inspection of the isolated peaks showed only a few examples of the peak splitting that would be expected to occur once all internal forks have terminated (as depicted in the last cartoon in Figure 7a). This suggests that even in the last time point (100–130 mins EdU) most Replicon Cluster Domains are still being replicated by internal forks in some cells in the population.

### Replication of valleys

**Figure 8.**
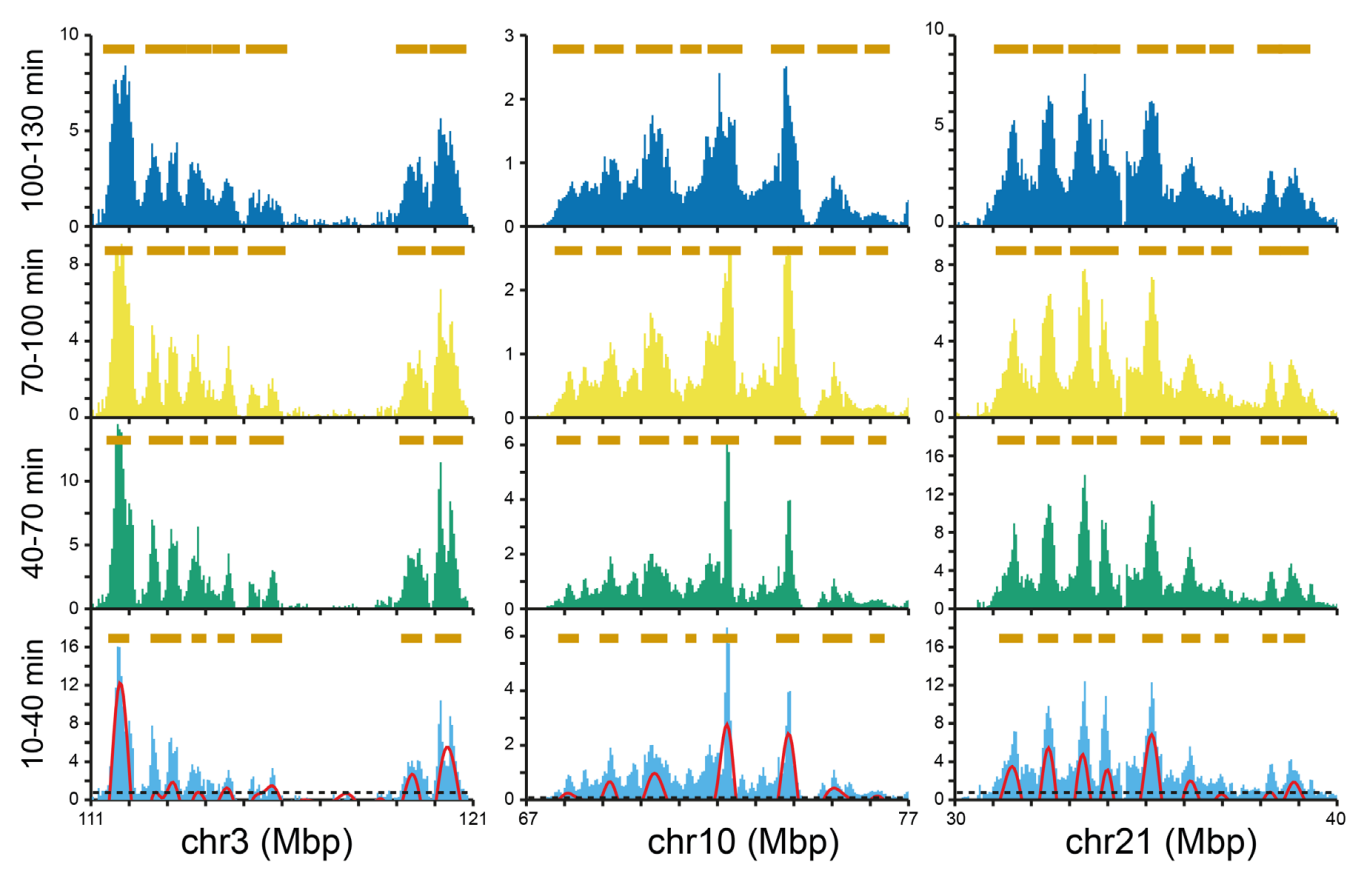
Examples of valley-filling between Replicon Cluster Domains. The early replication signal of three selected regions at the four time points is shown as light blue, green, yellow and dark blue bars (data in 50 kb bins). For the first time point (10-40 mins EdU) the wavelet analysis results with a wavelet peak width of 500 kb are shown by the red lines. The horizontal dashed line shows the cut-off for calling a wavelet peak as set at the 70 ^th^ percentile for peaks in late replicating domains. For the first time point, wavelet peaks are marked by horizontal brown bars; for the successive timepoints the edges of the bars were extended by 90 kb as expected of a fork moving at 1.5 kb/min.

We next considered the development of Replicon Cluster Domains packed together within large timing domains. Figure 8 shows three representative 10 Mbp segments that contain multiple Replicon Cluster Domains. The overall shape of the peaks was maintained over the two-hour time course, but there was a clear widening of the peaks as the valleys between the peaks became filled in. We considered whether this represents simple expansion of peaks due to the movement of its two flanking forks (as depicted in Figure 7a) or whether it is driven by new initiation events. Brown bars were drawn over peaks identified by wavelet analysis of the first time point and at successive time points these bars were lengthened by 90 kb (fork rate 1.5 kb/min).

The data show that in some regions where Replicon Cluster Domains are closely spaced the brown bars have merged by the last time point, consistent with the idea that some valley-filling could be accounted for by fork progression at the edges of clusters. However, many gaps still remain in the last time point so it is clear that this cannot account for replication of all the valley DNA. The median separation between Replicon Cluster Domains is ~1.2 Mbp ( Figure 5) and if they have a width of ~500 kb in size flanking forks progressing ~360 kb over the two-hour time course only extend the median cluster to a width of ~860 kb, which is two thirds of the distance required. This suggests that complete replication of valley DNA will depend on further initiation events. There is also good evidence for initiation in the valleys between Replicon Cluster Domains from the replication data ( Figure 2, Figure 3, Figure 8 and Underlying data: Supplementary Figure S3) ^51^ which shows that most valleys begin to replicate, albeit to a low level, even in the first time point. Because of the relatively high degree of synchrony obtained in these experiments ( Figure 1) we do not believe this ‘valley labelling’ is due to contaminating cells at a slightly later stage of S phase.

Instead, we favour the idea that there are active replication origins within these valleys. This would also be consistent with the considerable variation in the height of the peaks representing the Replicon Cluster Domains ( Figure 2 and Underlying data: Supplementary Figure S3) ^51^ which could be caused by them becoming active at different times in different cells in the population. Since DNA fibre analysis suggest that the majority of origins fire in clusters, our data would be consistent with the idea that ‘valleys’ between the prominent Replicon Cluster Domains also represent Replicon Cluster Domains that tend to become active at later times and which may also be invaded by forks emanating from neighbouring Replicon Cluster Domains that have become activated earlier in S phase.

**Figure 9.**
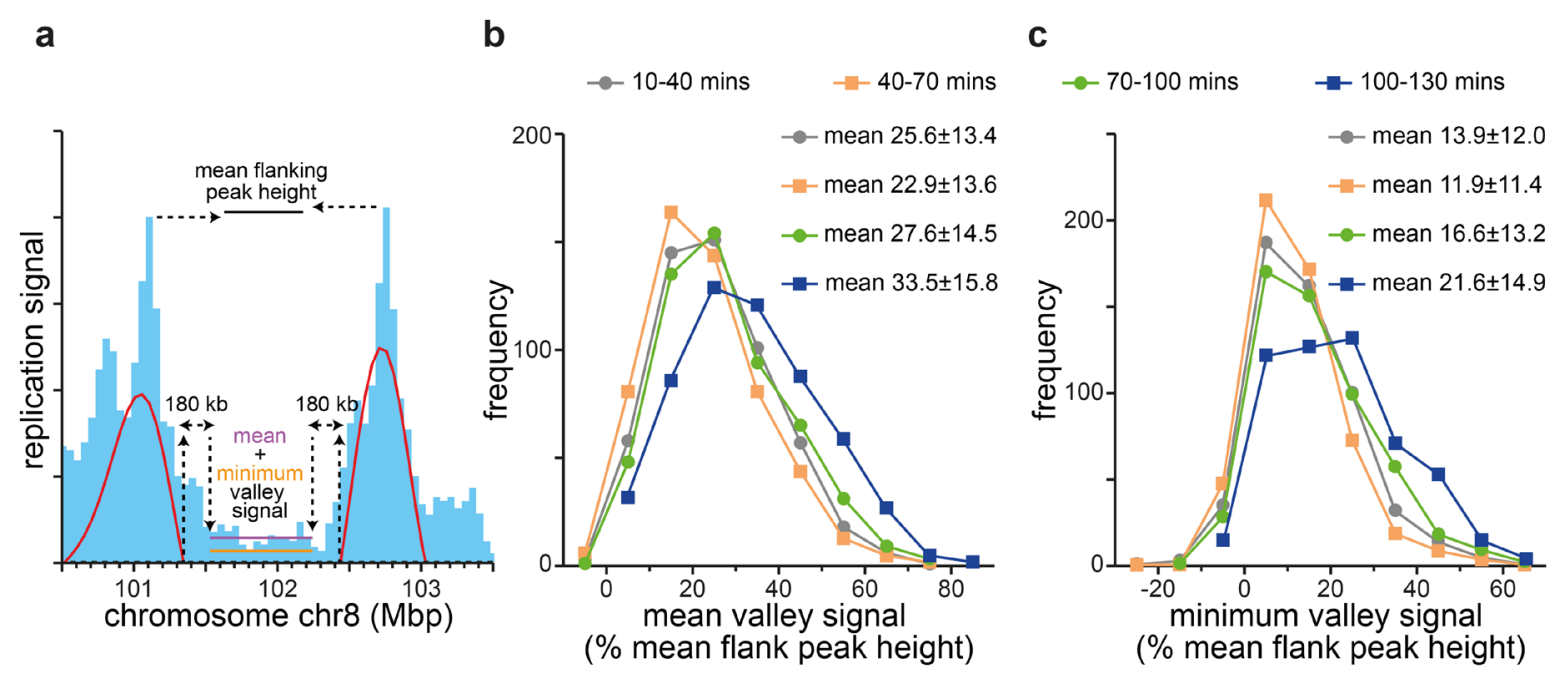
Analysis of valley-filling genome-wide. **a**) Schematic of how ‘valleys’ were analysed genome-wide, using a region on chromosome 8 as an example. The replication signal in the first time point (10–40 mins EdU; 50 kb bins) was subject to wavelet analysis with a wavelet peak width of 500 kb (red lines). For all pairs of wavelet peaks within any given early timing domain, the internal edges of the gap between the edges of the wavelet peaks were reduced by 180 kb to account for fork movement at 1.5 kb/min over two hours; the remaining gap was defined as the ‘valley’. Wavelet analysis with a peak width of 500 kb was then performed on the three later time points, and only those valleys whose flanking wavelet peaks existed (±100 kb error) in all four time points were included for further analysis. For each time point, the mean valley replication signal (horizontal purple line) and minimum valley replication signal (horizontal orange line) were expressed as a percentage of the mean height of the replication signals of the two flanking peaks (black horizontal line). **b**) The frequency distribution of the mean valley replication signal across the four time points. The mean and standard deviation of the distribution is also given. **c**) The frequency distribution of the minimum valley replication signal across the four time points. The mean and standard deviation of the distribution is also given.

To explore this idea further, we analysed the evolution of ‘valleys’ in a more systematic way. Figure 9a shows as an example a single valley on chromosome 8. We started with the 500 kb wavelet analysis of the first time point (10–40 mins EdU) though without any height cut-off for calling the peaks. As shown in Figure 9a, we defined ‘valleys’ as regions between two pairs of adjacent wavelet peaks residing in a single timing domain after we had added 180 kb to the edges of the wavelet peaks to account for fork movement (1.5 kb/min over 120 minutes). Six hundred and forty ‘valleys’ conformed to this definition in the first time point. We then examined the replication signal in the three later time points and rejected any valleys where the flanking peaks had shifted by more than 100 kb; this left 540 valleys that could be tracked across all four time points. Because the replication signal is not normalised between the different time points, we expressed the mean and minimum signal in each valley as a percentage of the mean height of the flanking peaks ( Figure 9a).

The frequency distribution of mean and minimum valley signals for the four timepoints are shown in Figures 9b and 9c respectively. Both the mean and minimum signal increases significantly in the last two time points. Importantly, by the last time point virtually every location in every valley has incorporated a significant amount EdU (525 out of 540 valleys have their minimum signal >10% mean flanking peak signal). This valley replication most likely represents new initiation events and is consistent with the idea that RCDs become activated right across the early timing domains, though with valley RCDs tend to activate later.

### Sequential activation of RCDs

There is experimental support for the idea that the different replicon clusters comprising a single timing domain are activated sequentially, the so-called domino model ^30, 52– 55^. In some DNA fibre studies that have analysed very long stretches of DNA, a second cluster has been shown to become active after a first cluster has been activated ^54^. In addition, dual labelling of replication foci shows evidence for a ‘domino’ activation model, where a second replication focus becomes activated immediately adjacent to a previously activated focus ^52, 53^. We therefore investigated whether we could see evidence for a domino-type activation of Replicon Cluster Domains in our early replication data; by this we mean that whether adjacent RCDs replicate sequentially at a frequency higher than random. A cursory look at the time course data ( Figure 2, Figure 3, Figure 8 and Underlying data: Supplementary Figure S3) ^51^ shows that Replicon Cluster Domains packed together in groups are not strictly arranged in order of height, indicating that there is no absolute ‘domino order’ by which they are activated. Nevertheless, we decided to investigate in a systematic way whether there is any evidence for a preferential activation order of adjacent Replicon Cluster Domains.

We first developed a metric to select ‘groups’ of adjacent Replicon Cluster Domains that might display some sort of activation order. Within individual timing domains, the mean spacing between Replicon Cluster Domains is 817 kb ( Figure 5b). We therefore defined adjacent Replicon Cluster Domains as being in the same ‘group’ if the distance between them was less than twice this value, i.e., <1.6 Mbp. The height of the peak was then defined as the maximum replication signal within the Replicon Cluster Domain, using the replication data mapped onto 50 kb bins. The activation order, as inferred from peak height, was analysed in two different ways. For small groups of three or four Replicon Cluster Domains, we examined every different permutation of peak height ranking. We next used an ‘adjacent height similarity’ metric to provide a global view of all groups of Replicon Cluster Domains.

**Figure 10.**
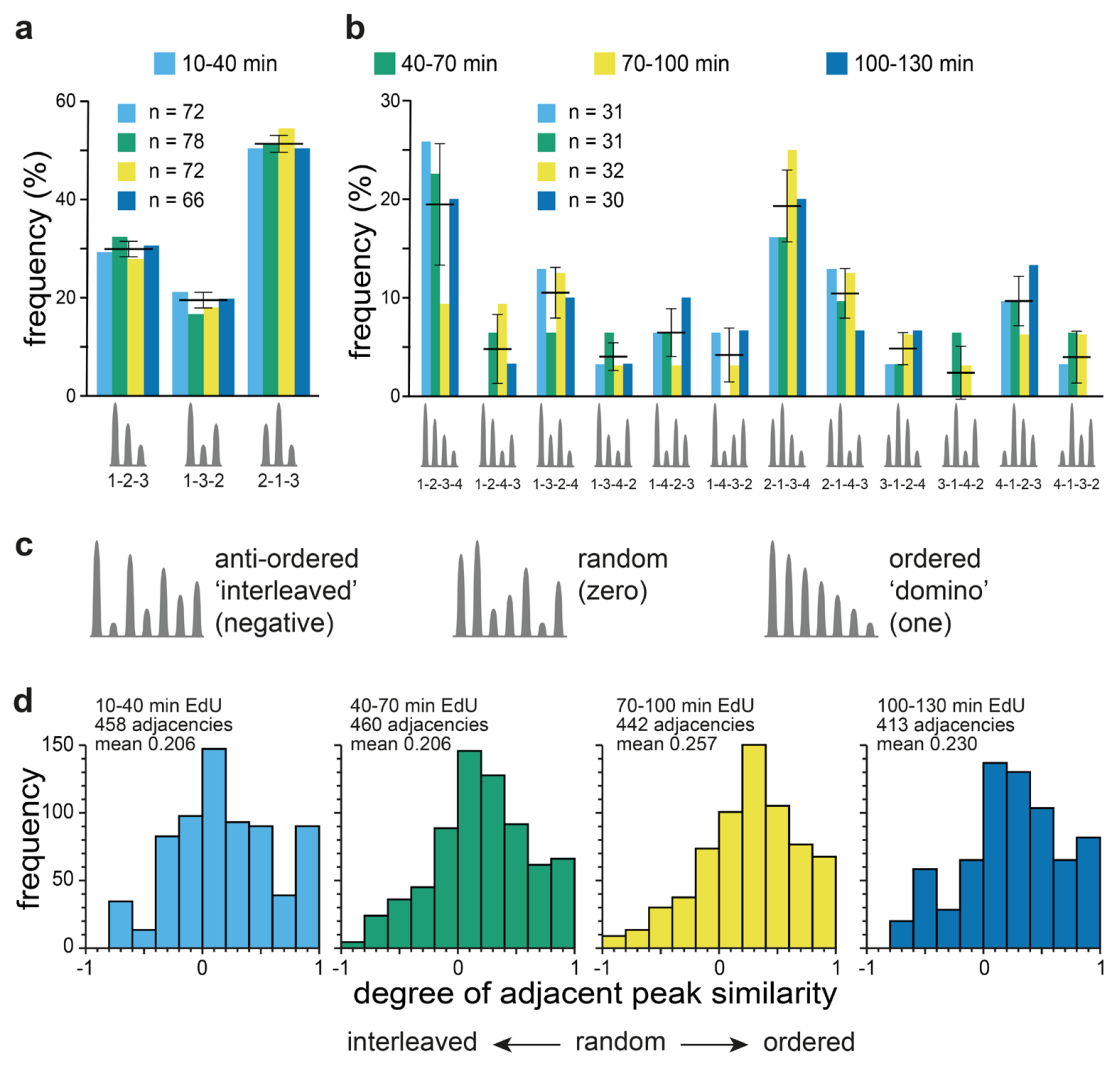
Activation order of Replicon Cluster Domains. The replication signal in the four time points (50 kb bins) was subject to wavelet analysis with a wavelet peak width of 500 kb. Wavelet peaks falling below the 70 ^th^ percentile of peaks identified in late replicating DNA were removed. The peak height of the replication signal within each wavelet peak was recorded. Groups of peaks were defined by being in the same timing domain and being less than 1600 kb apart. **a**) Analysis of groups with three members. Each peak was given a rank order dependent on the height of its replication signal and was classified into one of three possible height order permutations (1-2-3, 1-3-2 or 2-1-3). The percentage of groups with each activation sequence over the four time points is shown. Bars showing mean and standard deviation across the four timepoints are also shown. **b**) Analysis of groups with four members. Each peak was given a rank order dependent on the height of its replication signal and was classified into one of twelve possible height order permutations. The percentage of groups with each activation sequence over the four time points is shown. Bars showing mean and standard deviation across the four timepoints are also shown. **c**) Schematic showing possible examples of height order groups with 7 peaks. If adjacent peaks have maximal height differences, they will display an interleaved pattern and have a negative score in the adjacent peak similarity metric. If peak heights are randomly distributed they will on average have a zero score in the adjacent peak similarity metric. If peak heights are in perfect (‘domino’) order they will have a score of one in the adjacent peak similarity metric. **d**) Groups of peaks with four or more members were analysed by the adjacent peak similarity metric, and the frequency distribution in the four different time points is shown. Groups with three members were omitted from this analysis because with only three members the metric cannot distinguish between random and anti-ordered.

For groups containing three or four peaks we classified the height order of the peaks within each group, with the highest given a value of one, the next highest two and so on and then considered all the possible permutations of height order (bearing in mind that direction along the chromosome is arbitrary). The results are shown in Figure 10a (groups of three Replicon Cluster Domains) and 10b (groups of four Replicon Cluster Domains). All permutations are represented in the experimental data. However, some permutations are more abundant than others. For groups of three Replicon Cluster Domains there are three possible permutations with each having an expected frequency of 33.3% if the order was random. The data show a marked bias in the permutations, with the 2-1-3 ordering being the most abundant at 51.4 ± 1.7% across the four timepoints and the 1-3-2 ordering being the least abundant at 18.8 ± 1.6%. This bias towards the 2-1-3 ordering is consistent with there being a preference to activate a new Replicon Cluster Domain adjacent to a previously active one. For groups of four Replicon Cluster Domains there are twelve possible permutations with each having an expected frequency of 8.3% if the order was random. Again, the two most represented permutations are ones with the highest degree of height similarity: 1-2-3-4 (at 19.4 ± 16.2%) and 2-1-3-4 (at 19.3 ± 13.6%). These results are consistent with a ‘domino’ activation sequence being preferred though not strictly necessary.

To test this against all group sizes, we devised an ‘adjacent height similarity’ metric which reported whether the peaks were in perfect height order (‘domino’) with a value of 1, randomly ordered with a value of 0, or avoided adjacent height similarity (‘interleaved’) with a negative value ( Figure 10c). This metric was calculated for all groups of Replicon Cluster Domains containing four or more members and the distributions are shown in Figure 10d. At all four timepoints there was a clear quantitative preference for peaks of similar sizes to lie next to one another. It is not possible to tell from the population data whether this preference is seen within individual cells or whether it is only a feature of the population as a whole. However, the result is consistent with the idea that whilst the activation timing of Replicon Cluster Domains is variable in the population, within individual cells the presence of an active Replicon Cluster Domain enhances the probability that an adjacent Replicon Cluster Domain subsequently becomes active.

## Discussion

We provide here the first evidence that we are aware of for the existence of Replicon Cluster Domains at a DNA sequence level. We have labelled and sequenced replicating DNA from cells passing through the early stages of S phase at a relatively high degree of synchrony compared to previous studies. The replication signal in our data is consistent with replication timing data from cells at a lower degree of synchrony, but our data show a much higher degree of fine-scale structure. Our results provide a bridge between results obtained by DNA fibre analysis and microscopy with mapped genomic loci.

Although the existence of replicon clusters – adjacent groups of synchronously firing origins – has been known for a very long time, the genomic sequences that they correspond to have remained unknown. We report here that early replicating DNA shows a broad range of peaks at specific and consistent genomic locations. The genomic locations of these early replicating peaks are highly consistent with previous results using a lower temporal resolution and fall within regions previously identified as Early Timing Domains ^22, 26, 27^. However, our results show much more fine-scale structure than was shown by previous studies analysing the structure of Replication Timing Domains. This is consistent with the idea that individual Replication Timing Domains consist of a collection of smaller domains that become active at slightly different times, either early or late in S phase ^22, 30^. It has previously been suggested that Replication Timing Domains consist of smaller functional units because the locations of Replication Timing Domains vary between different cell types, and differences in Timing Domain location tend to divide them at reproducible positions.

Wavelet analysis of the early replicating DNA shows that the maximum signal for these peaks is 500 kb, almost exactly as would be expected from quantitative analysis of DNA fibre analysis of replicon clusters ^8, 15, 54^. As cells progress through S phase, the width of these peaks grows at rates consistent with the progression of a single pair of replication forks at the extreme edges of each peak. We therefore propose that these peaks represent the DNA synthesised by individual replicon clusters and have named their genomic locations ‘Replicon Cluster Domains’. Since there is widespread speculation that replicon clusters might correspond to replication foci ^8, 15, 30, 36^, Replicon Cluster Domains might also correspond to the genomic positions where replication foci are active early in S phase. Boundaries between Replicon Cluster Domains might also represent the boundaries between Topologically Associated Domains which have been shown to represent the borders between replication timing domains conserved between different cell lines ^23, 56^.

One striking feature of the peaks of early replicating DNA is an extreme variability in their heights spanning a >20-fold difference. Whilst some degree of variation in the intensity of the replication signal would arise from differences in the density of active replication origins, this degree of difference in origin density has not been observed by classical DNA fibre analysis where individual DNA fibres are selected and individually analysed ^8, 15^. However, our results appear to be consistent with the extreme stochasticity recently observed using a high-throughput single-molecule approach ^7^. The alternative explanation for the height differences is that that there is some stochasticity in the time that Replicon Cluster Domains become active, and the height of a peak reflects the number of cells in the population that have an actively replicating cluster at that particular location. By this interpretation, which we favour, high peaks represent Replicon Cluster Domains that are efficiently activated early in S phase, and low peaks represent Replicon Cluster Domains that are inefficiently activated early in S phase and/or typically become activated only later in S phase.

Replicon Cluster Domains are packed fairly tightly into early replicating DNA with mean spacing of 1.2 Mb across all early replication timing domains. However, there is no regularity in this spacing, consistent with the idea that Replicon Cluster Domains vary in size and can become active at different times in different cells in the population. This conclusion is also supported by examination of the replication of the valleys between the early replicating peaks. In the two-hour time course we examined, only a few of the valleys could be completely replicated by the forks from the edges of the flanking peaks, suggesting the need for further initiation events to complete replication. Consistent with this idea, we see the replication signal building up with time at locations too far from the flanking peaks to be accounted for by replication forks progressing outwards from the peaks. The mean spacing of 1.2 Mb early-firing Replicon Cluster Domains is consistent with the presence of Replicon Cluster Domains in the valley bottoms that are activated later in S phase. Taken together, our data suggest that initiation takes place within the valley bottoms to promote replication of all the DNA in the early replicating domains.

We also examined whether there was any discernible pattern in the heights of adjacent peaks that might reflect features affecting the time or efficiency with which they became active. Although there was substantial a variability in the peak height order, we showed that there was a marked tendency for adjacent peaks to have similar heights. This is consistent with a ‘domino’-style model where the presence of one active replicon cluster increases the probability that a neighbouring replicon cluster becomes active ^19, 30, 52– 55^. The mechanism that leads to the preference for domino-style activation remains to be determined, but one can speculate that it depends on the chromatin context within which each Replicon Cluster Domain is situated.

The results presented here give one of the first glimpses of how replication might be organised at the sub-megabase level in somatic metazoan cells, potentially integrating at genomic loci previously disparate results obtained by DNA fibre analysis and high-resolution microscopy. Our results show the potential for mapping DNA replication at a very high temporal resolution. Because cell cycle synchrony is gradually decreased as our cohort of synchronised cells progress through S phase, the technique described here can only be used to analyse the early stages of S phase at high temporal resolution. To further explore these possibilities, technical refinements might be required for achieving higher temporal resolution. Isolating cells that are at very precise stages of S phase is difficult, and the use of alternative possibilities that do not require cell cycle synchronisation might be a better approach. One possibility would be to sort cells using very fine differences in DNA content. Another possible approach would be to reconstruct the timing programme from the analysis of sites of EdU incorporation in individual S phase cells ^7, 28, 29, 57– 59^.

However, despite some drawbacks that are associated with the cell cycle synchrony we have used, this paper reports data that is unbiased and does not depend on assumptions about the structure of S phase. This high-resolution mapping of ours provides new insights into the dynamics and organisation of the genome for duplication.

## Conclusions

We show that early replicating DNA is organised into Replicon Cluster Domains that behave as expected of replicon clusters observed by DNA fibre analysis. The domains have a range of sizes that cluster around 500 Mbp. The coordinated activation of different Replicon Cluster Domains can generate the replication timing programme by which the genome is duplicated. Different Replicon Cluster Domains show marked differences in their labelling intensity and we provide evidence that this is at least in part part caused by Replicon Cluster Domains being activated at different times in different cells in the population. We also provide evidence that adjacent clusters were preferentially activated in sequence across a group, consistent with the ‘domino’ model of replication focus activation observed by microscopy.

## Data Availability

BioStudies: Underlying data for ‘The location and development of Replicon Cluster Domains in early replicating DNA’. https://www.ebi.ac.uk/biostudies/studies/S-BSST966 ^51^ This project contains the following underlying data: E1_1_1.fastq.gz (Control, 0-40 min, treatment, replicate 1) E1_1_2.fastq.gz (Control, 40-70 min, treatment, replicate 1) E1_1_3.fastq.gz (Control, 70-100 min, treatment, replicate 1) E1_1_4.fastq.gz (Control, 100-130 min, treatment, replicate 1) E1_1_5.fastq.gz (Control, 0-130 min, treatment, replicate 1) E1_PD_1.fastq.gz (Pulldown, 0-40 min, treatment, replicate 1) E1_PD_2.fastq.gz (Pulldown, 40-70 min, treatment, replicate 1) E1_PD_3.fastq.gz (Pulldown, 70-100 min, treatment, replicate 1) E1_PD_4.fastq.gz (Pulldown, 100-130 min, treatment, replicate 1) E1_PD_5.fastq.gz (Pulldown, 0-130 min, treatment, replicate 1) E2_1_1.fastq.gz (Control, 10-40 min, treatment, replicate 2) E2_1_10.fastq.gz (Control, 0-130 min, treatment, replicate 3) E2_1_11.fastq.gz (Control, 10-40 min, treatment, replicate 4) E2_1_12.fastq.gz (Control, 10-40 min, treatment, replicate 5) E2_1_13.fastq.gz (Control, 40-70 min, treatment with hydroxyurea, replicate 2) E2_1_14.fastq.gz (Control, 70-100 min, treatment with hydroxyurea, replicate 3) E2_1_15.fastq.gz (Control, 0-30 min, asynchronous, replicate 4) E2_1_17.fastq.gz (Control, 0-60 min, asynchronous unsorted, replicate 1) E2_1_2.fastq.gz (Control, 40-70 min, treatment, replicate 2) E2_1_20.fastq.gz (Control, 0-40 min, treatment, replicate 1) E2_1_21.fastq.gz (Control, 0-40 min, treatment, replicate 2) E2_1_22.fastq.gz (Control, 0-40 min, treatment, replicate 3) E2_1_23.fastq.gz (Control, 0-30 min, asynchronous, replicate 1) E2_1_24.fastq.gz (Control, 0-30 min, asynchronous, replicate 2) E2_1_25.fastq.gz (Control, 0-30 min, asynchronous, replicate 3) E2_1_26.fastq.gz (Control, 10-40 min, treatment with hydroxyurea, replicate 1) E2_1_3.fastq.gz (Control, 70-100 min, treatment, replicate 2) E2_1_4.fastq.gz (Control, 100-130 min, treatment, replicate 2) E2_1_5.fastq.gz (Control, 0-130 min, treatment, replicate 2) E2_1_6.fastq.gz (Control, 10-40 min, treatment, replicate 3) E2_1_7.fastq.gz (Control, 40-70 min, treatment, replicate 3) E2_1_8.fastq.gz (Control, 70-100 min, treatment, replicate 3) E2_1_9.fastq.gz (Control, 100-130 min, treatment, replicate 3) E2_PD_1.fastq.gz (Pulldown, 10-40 min, treatment, replicate 2) E2_PD_10.fastq.gz (Pulldown, 0-130 min, treatment, replicate 3) E2_PD_11.fastq.gz (Pulldown, 10-40 min, treatment, replicate 4) E2_PD_12.fastq.gz (Pulldown, 10-40 min, treatment, replicate 5) E2_PD_13.fastq.gz (Pulldown, 40-70 min, treatment with hydroxyurea, replicate 2) E2_PD_14.fastq.gz (Pulldown, 70-100 min, treatment with hydroxyurea, replicate 3) E2_PD_15.fastq.gz (Pulldown, 0-30 min, asynchronous, replicate 4) E2_PD_17.fastq.gz (Pulldown, 0-60 min, asynchronous unsorted, replicate 1) E2_PD_2.fastq.gz (Pulldown, 40-70 min, treatment, replicate 2) E2_PD_20.fastq.gz (Pulldown, 0-40 min, treatment, replicate 1) E2_PD_21.fastq.gz (Pulldown, 0-40 min, treatment, replicate 2) E2_PD_22.fastq.gz (Pulldown, 0-40 min, treatment, replicate 3) E2_PD_23.fastq.gz (Pulldown, 0-30 min, asynchronous, replicate 1) E2_PD_24.fastq.gz (Pulldown, 0-30 min, asynchronous, replicate 2) E2_PD_25.fastq.gz (Pulldown, 0-30 min, asynchronous, replicate 3) E2_PD_26.fastq.gz (Pulldown, 10-40 min, treatment with hydroxyurea, replicate 1) E2_PD_3.fastq.gz (Pulldown, 70-100 min, treatment, replicate 2) E2_PD_4.fastq.gz (Pulldown, 100-130 min, treatment, replicate 2) E2_PD_5.fastq.gz (Pulldown, 0-130 min, treatment, replicate 2) E2_PD_6.fastq.gz (Pulldown, 10-40 min, treatment, replicate 3) E2_PD_7.fastq.gz (Pulldown, 40-70 min, treatment, replicate 3) E2_PD_8.fastq.gz (Pulldown, 70-100 min, treatment, replicate 3) E2_PD_9.fastq.gz (Pulldown, 100-130 min, treatment, replicate 3) Supp Figure S1.pdf (Supplemental Figure S1) Supp Figure S2.pdf (Supplemental Figure S2) Supp Figure S3.pdf (Supplemental Figure S3) Supp Figure S4.pdf (Supplemental Figure S4) Supp Figure S5 v3.pdf (Supplemental Figure S5) Data are available under the terms of the Creative Commons Zero “No rights reserved” data waiver (CC0 1.0 Public domain dedication). 4D Nucleome: Late fraction S-phase Repliseq on U2OS Tier 2 cells [Homo sapiens]. Accession number 4DNES99LXRYK; https://identifiers.org/4dn:4DNES99LXRYK 4D Nucleome: Early fraction S-phase Repliseq on U2OSTier 2 cells [Homo sapiens]. Accession number 4DNES1P18J2X; https://identifiers.org/4dn:4DNES1P18J2X

## Software availability

Source code available from: https://github.com/bartongroup/MG_EarlyReplication

Archived source code at time of publication: https://www.doi.org/10.5281/zenodo.7639072 ^41^

License: CC0
