# Supplementary material for: The location and development of Replicon Cluster Domains in early replicating DNA
Source: Wellcome Open Res. 2023 Aug 22;8:158. Originally published 2023 Apr 11. [Version 2] doi: 10.12688/wellcomeopenres.18742.2 (PMC10521077; doi:10.12688/wellcomeopenres.18742.2)
Supplement: Peer review history

# Reviewer report: Reviewer response for version 2

**DOI:** 10.21956/wellcomeopenres.22056.r65746

**Published:** 2023-09-29

**Authors:** Zhongqing Ren

**Affiliations:** 1 Indiana University Bloomington, Bloomington, Indiana, USA

The authors have addressed my concerns with the original manuscript.

Is the work clearly and accurately presented and does it cite the current literature?

Yes

If applicable, is the statistical analysis and its interpretation appropriate?

Not applicable

Are all the source data underlying the results available to ensure full reproducibility?

Yes

Is the study design appropriate and is the work technically sound?

Partly

Are the conclusions drawn adequately supported by the results?

Partly

Are sufficient details of methods and analysis provided to allow replication by others?

Yes

Reviewer Expertise:

Chromosome organization and segregation

I confirm that I have read this submission and believe that I have an appropriate level of expertise to confirm that it is of an acceptable scientific standard.

# Reviewer report: Reviewer response for version 2

**DOI:** 10.21956/wellcomeopenres.22056.r65747

**Published:** 2023-09-07

**Authors:** Itamar Simon, Avraham Greenberg

**Affiliations:** 1 Department of Microbiology and Molecular Genetics, IMRIC, Faculty of Medicine, Hebrew University of Jerusalem, Jerusalem, Israel

In light of the authors’ answers, and the changes that they have made, I would give the revised article the status of 'approved', and I thank the authors for addressing my concerns.

Is the work clearly and accurately presented and does it cite the current literature?

Yes

If applicable, is the statistical analysis and its interpretation appropriate?

Partly

Are all the source data underlying the results available to ensure full reproducibility?

Yes

Is the study design appropriate and is the work technically sound?

Yes

Are the conclusions drawn adequately supported by the results?

Yes

Are sufficient details of methods and analysis provided to allow replication by others?

Yes

Reviewer Expertise:

NA

We confirm that we have read this submission and believe that we have an appropriate level of expertise to confirm that it is of an acceptable scientific standard.

# Reviewer report: Reviewer response for version 2

**DOI:** 10.21956/wellcomeopenres.22056.r65748

**Published:** 2023-09-07

**Authors:** Ichiro Hiratani

**Affiliations:** 1 RIKEN Center for Biosystems Dynamics Research, Kobe, Japan

Is the work clearly and accurately presented and does it cite the current literature?

Yes

If applicable, is the statistical analysis and its interpretation appropriate?

I cannot comment. A qualified statistician is required.

Are all the source data underlying the results available to ensure full reproducibility?

Yes

Is the study design appropriate and is the work technically sound?

Yes

Are the conclusions drawn adequately supported by the results?

Yes

Are sufficient details of methods and analysis provided to allow replication by others?

Yes

Reviewer Expertise:

NA

I confirm that I have read this submission and believe that I have an appropriate level of expertise to confirm that it is of an acceptable scientific standard.

# Reviewer report: Reviewer response for version 1

**DOI:** 10.21956/wellcomeopenres.20781.r60829

**Published:** 2023-07-18

**Authors:** Zhongqing Ren

**Affiliations:** 1 Indiana University Bloomington, Bloomington, Indiana, USA

In this study, the authors employed EdU pull-down combined with high-throughput sequencing on synchronized human cells to study the replication cluster domains in early S phase. This whole-genome scale method complements DNA fiber analysis, which analyzes individual molecules.

 The results are consistent with the previous studies and revealed more details at fine-scale about the early replication cluster domains. Taking advantage of the higher resolution of the data, the authors defined the sizes, spacing and expansion rates of the replication cluster domains.

 Furthermore, the authors provided evidence supporting that late-activated replication cluster domains could fill the "gaps" between the early-activated replication cluster domains, and a "domino" activation model where the early-fired replication cluster domains increase the probability of the activation of the adjacent replication cluster domains. Overall, the experiments were well controlled and the data were nicely analyzed. Although the cells were not well synchronized, the article still provides valuable and interesting information. I do not request more experiments but have a few comments:

**Major:**

1. From Figure S1, one can see that the cells were not synchronized very well. Even after FCAS sorting and selection, some chromosome regions have higher copy number than the other (Figure S2), suggesting some regions may have already started replication. The authors should consider the effects of the pre-fired origins when analyze, interpret and discuss the results. For the future experiments, the authors may need to optimize their synchronization protocol (e.g., double thymidine block (Chen et al., 2018 ^1^)) or use a different synchronization method (e.g., CDKs inhibition).

**Minor:**

1. " *This increase in DNA content can be seen in synchronised cells pulsed continuously with EdU for 130 min (Figure 1b, 0–130 min).*" It is hard to see the increase in whole DNA content in Fig 1b. It would be helpful to provide some quantified results.
2. It would be helpful if the authors can explain why the Ricker wavelet is used for analyzing this type of data. 
3. The color scales for the heatmaps are absent in Figure 2C and 3. The color contrast is not great. May want to consider to change the color scheme. 
4. " *This is in line with the two later expansion rates*". The expansion between the 2nd and 3rd time points is not really in line with 60-90kb. There is an increase of expansion rate for these four time points, which is better to be mentioned and discussed.
5. " *The median separation between Replicon Cluster Domains is ~1.2 Mbp (Figure 5) and if they have a width of ~500 kb in size flanking forks progressing ~360 kb over the two-hour time course only extend the median cluster to a width of ~860 kb, which is two thirds of the distance required. This suggests that complete replication of valley DNA will depend on further initiation events.*". This argument is not strong. The separated replication cluster domains might fill the entire valley after three hours. Additional information is needed to justify the statement, e.g., the replication forks usually stay on the chromosome for xx hours. 
6. " *There is also good evidence for initiation in the valleys between Replicon Cluster Domains from the replication data*". It is hard to find the related region in these figures. It will be helpful to use arrows to point out the related regions. 
7. " *This would also be consistent with the considerable variation in the height of the peaks representing the Replicon Cluster Domains which could be caused by them becoming active at different times in different cells in the population.*" My understanding is that replication cluster domains with the lower height of peaks generally activate later than those with higher peaks in the population. It that correct? If so, the sentence quoted above is not accurate and need to be revised.
8. For Figure S2, more details about how each control was processed are needed. What is the difference between 1% and 99% samples of the G1 FACS-sorted cells?

Is the work clearly and accurately presented and does it cite the current literature?

Yes

If applicable, is the statistical analysis and its interpretation appropriate?

Not applicable

Are all the source data underlying the results available to ensure full reproducibility?

Yes

Is the study design appropriate and is the work technically sound?

Partly

Are the conclusions drawn adequately supported by the results?

Partly

Are sufficient details of methods and analysis provided to allow replication by others?

Yes

Reviewer Expertise:

Chromosome organization and segregation

I confirm that I have read this submission and believe that I have an appropriate level of expertise to confirm that it is of an acceptable scientific standard, however I have significant reservations, as outlined above.

## References

[1] : Cell Synchronization by Double Thymidine Block. *Bio Protoc* .2018;8(17) : 10.21769/BioProtoc.2994 10.21769/BioProtoc.2994 30263905PMC6156087

# Response

**Published:** 2023-08-14

**Authors:** J Julian Blow

**Affiliations:** University of Dundee, UK

**Major:**

1. *From Figure S1, one can see that the cells were not synchronized very well. Even after FCAS sorting and selection, some chromosome regions have higher copy number than the other (Figure S2), suggesting some regions may have already started replication. The authors should consider the effects of the pre-fired origins when analyze, interpret and discuss the results. For the future experiments, the authors may need to optimize their synchronization protocol (e.g., double thymidine block (Chen et al., 2018 ^1^ )) or use a different synchronization method (e.g., CDKs inhibition).*
  Achieving the best possible synchronisation procedure was one of the major challenges of this work. Our protocol allowed a significantly tighter burst of S phase entry compared to standard methods such as double thymidine synchronisation. Despite our best efforts, entry into S phase was staggered over a period of about 45 minutes as shown in Figures 1, S1 and S2. There is little evidence to suggest that cells leaked through the mimosine block as suggested by the reviewer. Figure S2 shows increasing pulses of EdU incorporation after mimosine release (0, 2.5, 5, 10, 15, 20, 25, 30 and 40 minutes after mimosine release). Virtually no EdU incorporation is seen for the first 10 minutes after mimosine release and provides evidence that the vast majority of cells incorporating EdU do so from a non-replicating G1 DNA content. There are a relatively small number of cells that incorporate EdU with a mid- to late- S phase DNA content, indicative of cells that didn’t complete the previous S phase after thymidine release. Almost all of these cells were lost following the FACS sort for cells with a near-G1 DNA content. For a traditional block-and-release synchronisation procedure we think that these cells were synchronised very well. However, we agree that alternative technical approaches, such as are mentioned at the end of the paper, would improve the synchrony still further.  

**Minor:**

1. *"This increase in DNA content can be seen in synchronised cells pulsed continuously with EdU for 130 min (Figure 1b, 0–130 min)." It is hard to see the increase in whole DNA content in Fig 1b. It would be helpful to provide some quantified results.*
  For all pulses where EdU incorporation is evident in Figure 1 and Figure S1 (i.e. pulses longer than 15 min), the EdU-labelled DNA very clearly moves to the right on the x-axis away from the peak of G1 cells, representing an increase in total DNA content. To make this more obvious we have replotted the FACS data in Figure 1 and Supplementary Figure S1 to decrease the extent of the y axis (DNA content) thereby making the increase in DNA content more obvious. We also have amended the above text in the Results section to describe this: “ *This increase in DNA content can be seen in synchronised cells pulsed with EdU for 100-130 min (Figure 1b), where the total increase in DNA content is ~15%; shorter pulses of EdU (Underlying data: Supplementary Figure S1) also show increases in DNA content, with ~6% increase in DNA content after 40 min. This slightly slowed progression through S phase could be due to mimosine inducing a small number of double strand breaks (47-50) or reducing cellular dNTP pools (52).*” Please also see response to Reviewer 2’s second major point.
2. *It would be helpful if the authors can explain why the Ricker wavelet is used for analyzing this type of data.*
  The Ricker wavelet is one of the most basic wavelets used for analysis and is more likely to approximate our data than other more complex wavelets. We have added the following sentence to the Results section when we introduce the wavelet analysis: “ *We chose to use a Ricker wavelet (Supplementary Figure S4) for the analysis because it is a simple symmetrical wavelet that is derived from a Gaussian distribution and therefore makes minimal assumptions about the expected shape of the peaks.*”
3. *The color scales for the heatmaps are absent in Figure 2C and 3. The color contrast is not great. May want to consider to change the color scheme. *
  We have added a heatmap colour scheme to the Figure and added a sentence to the legend saying: “Dark blue represents a signal of zero or below, and red represents a signal of one.” The relative lack of orange and red in Figure 2 is because the 500 kb peaks are extremely small at this scale; they only become apparent after zooming in to smaller regions as in Figure 3.
4. *"This is in line with the two later expansion rates". The expansion between the 2nd and 3rd time points is not really in line with 60-90kb. There is an increase of expansion rate for these four time points, which is better to be mentioned and discussed.*
  As requested by Reviewer 2, we have added some more statistical information to this figure (panel 7G). We have also recalculated the rate information in panels 7E and 7F in kb/min for better comparison with 7G. This shows that between the 2 ^nd^ and 3 ^rd^ time points, the width increases by 1.3 kb/min (wavelet analysis) and 0.37 kb/min (Gaussian analysis), which we agree is on the low side for two forks. We have therefore changed the phrase to " *This is in line with the later expansion rates*".
5. *"The median separation between Replicon Cluster Domains is ~1.2 Mbp (Figure 5) and if they have a width of ~500 kb in size flanking forks progressing ~360 kb over the two-hour time course only extend the median cluster to a width of ~860 kb, which is two thirds of the distance required. This suggests that complete replication of valley DNA will depend on further initiation events.". This argument is not strong. The separated replication cluster domains might fill the entire valley after three hours. Additional information is needed to justify the statement, e.g., the replication forks usually stay on the chromosome for xx hours. *
  The reviewer is correct that the assumption of the sentence was about replication within the period of the analysis. For greater clarity we have therefore changed the sentence to: “This suggests that complete replication of valley DNA within a two hour period would depend on further initiation events.”
6. *"There is also good evidence for initiation in the valleys between Replicon Cluster Domains from the replication data". It is hard to find the related region in these figures. It will be helpful to use arrows to point out the related regions. *
  The examples in Figure 8 are worked through, with the brown bars showing expected fork progression. In case this isn’t clear enough we have amended the sentence describing this (as underlined) to: “ *However, many gaps between the brown bars still remain in the last time point so it is clear that this cannot account for replication of all the valley DNA*.”
7. *"This would also be consistent with the considerable variation in the height of the peaks representing the Replicon Cluster Domains which could be caused by them becoming active at different times in different cells in the population." My understanding is that replication cluster domains with the lower height of peaks generally activate later than those with higher peaks in the population. It that correct? If so, the sentence quoted above is not accurate and need to be revised.*
  Yes the reviewer’s interpretation corresponds to what we were trying to convey, though the text was perhaps not so clear. To clarify, we have amended the sentence to: “This would also be consistent with the considerable variation in the height of the peaks representing the Replicon Cluster Domains … which could be caused by them becoming active at different times, with lower peak heights representing Domains that tend to become active at later times.”
8. *For Figure S2, more details about how each control was processed are needed. What is the difference between 1% and 99% samples of the G1 FACS-sorted cells?*
  The processing and normalisation of the data is described in quite some detail in Methods under the headings “Se *quencing data processing” and “Background subtraction and normalisation”.* For the difference between the 1% and 99% data (which was unexpected), we have added the following sentence to the legend to Figure S2 to explain why we think the last three samples (including the G1_99 sample) are much less uniform than the others: “We believe that the more erratic profiles of the last three samples is due to the fact that considerably more DNA was submitted for library preparation, which resulted in competition between DNA and limiting amounts of primer.” We did not use these last three data sets for normalisation.

# Reviewer report: Reviewer response for version 1

**DOI:** 10.21956/wellcomeopenres.20781.r56116

**Published:** 2023-05-03

**Authors:** Itamar Simon, Avraham Greenberg

**Affiliations:** 1 Department of Microbiology and Molecular Genetics, IMRIC, Faculty of Medicine, Hebrew University of Jerusalem, Jerusalem, Israel

In this paper the authors used a combination of synchronization, EdU pulses and pulldown, and careful normalization and wavelet analysis in order to visualize the replication of origin clusters at a genomic level in early S phase. The detailed and in-depth bioinformatics in this paper managed to address a few aspects of replication organization, which have previously mainly been addressed by DNA fiber analyses. This includes the observations that:

1. replication domains are built of several replicons that fire simultaneously, which was previously deduced by a combination of replication domain sizes (genomics) and replicon sizes (DNA combing);
2. replication domains extend in both directions by single forks moving in each direction at the ends of the domains (this is the common interpretation of the TTR that usually flanks replication domains);
3. expansion of domains by TTR is not sufficient to fill the gaps between early domains thus one must assume the initiation of later domains (this is the common interpretation of replication maps);
4. early domains somehow promote the activation of adjacent later domains (the domino model that was previously suggested was based on microscopy and DNA fiber analyses).

Overall, the work is an elegant application of a number of analytic techniques to biological questions, which were previously studied using mainly DNA combing techniques. Thus, the findings in this paper are not new, but provide new support for previously published findings using a different type of technology.

**Major points:**

- The authors have not demonstrated why EdU immune-precipitation in early S phase cells provides higher resolution than Repli-seq of non-synchronized cells. Would similar wavelet analysis of Repli-seq data not also show similar trends? If the aim is to demonstrate that domains are replicon clusters, couldn't this be done by analyzing Repli-seq data without the need to synchronize the cells and pulldown with EdU?
- We are surprised not to see substantial progress along the cell cycle (i.e., PI intensity) even after 2 hours. Did the authors take any timepoints (for **Figure 1B/S1**) later than 2 hours? Could the authors add quantification (for example using gates) to show the progress along the x-axis in **Figure 1B**? The lack of progression raises a question about how representative the results are; do they reflect a normal unperturbed cell cycle or do they represent a specific case in which the entry into S phase is very slow.

**Minor points:**

1. In the second paragraph of the Introduction the authors state " *Indeed, of the total number of potentially available replication origins present in diploid mammalian cells – roughly 100,000,000 – only approximately 10% will be used to initiate DNA replication*". Is the 100,000,000 a typo? We are more familiar with lower estimates, specifically 500,000 licensed origins.
2. The source for the RT data is provided as https://www2.replicationdo-main.com/database.php, but this link is not accessible. As such the data is currently not accessible to the public.
3. The inset in **Figure 1B** is not explained.
4. **Figure 2C** - scale of graph missing (and explanation of the units of the score)
5. **Reference 48** seems the wrong reference for page 9 (" *This is consistent with recent data on single DNA fibres which shows the high degree of stochasticity that occurs in origin firing*").
6. **Figure 4A **and **B** – What does the 0-40 timepoint add over the 10-40 timepoint? Also why is the 10kb and the 50kb comparison important? Also, 4c and 4d come to justify the 70 percentile cutoff, but this has already been relied on in **Figure** **3**. Perhaps the figure order could be swapped between **Figures 3** and **4**? Or **4** could be moved to supplementary.
7. When discussing **Figure 4D**, page 12, the authors say that the variance between the clusters is due to stochasticity between cells. We can think of 2 alternative explanations. Based on what we have observed in published data, and in our own lab's unpublished data, inter-origin distance has as much variance as the 20-fold variance shown in **Figure 4D**. Alternatively, the variance could reflect locus to locus variation in the time that different Replicon Cluster Domains become active. Although these three options are discussed in the discussion it might help to mention all three in the results as well, as the conclusion that stochasticity between cells is responsible remains not fully proven.
8. **Figure 5B** – how the early replication domain are defined?
9. Regarding the Isolated Replicon Cluster Domains:
   
  1. **Figures 7C-F** are based on only 72 isolated domains. How sure are they that the conclusions derived from the isolated domains are relevant to more crowded regions?
  2. The authors say " *We rejected from further analysis 51 peaks*" was there a biological justification for removing these, instead of expanding the range of widths (e.g, 100-1500).
10. Statistical tests are missing in **figures 7E-F, 9B-C** and **10**. 
11. In my understanding the edges of the timing domains reflect what is generally referred to as TTR. The authors should mention this, for example when discussing the valley filling in **Figure 8**, or in the discussion " *As cells progress through S phase, the width of these peaks grows at rates consistent with the progression of a single pair of replication forks at the extreme edges of each peak*.".
12. In **Figure 8**, the expansion of the bars should start from the 40-70m timepoint in line with what the authors wrote on page 14 (" *The lack of significant growth between the first two time points …*").
13. Near the end of the results the authors say " *the mean spacing is 817 kb"*. This appears to be a mistake, based on **Figure 5** (0.817 is the number of peaks per Mbp. The real number should be 1.2 Mb spacing between peaks). The rest of the analysis of the adjacent Replicon Cluster Domains for **Figure 10** should be recalculated accordingly.
14. **Figures 10C** and **D** use an elegant metric but do not accurately measure the domino effect, as they assume that the outermost origin must be the first one to be activated. For example, by this logic, the 2-1-3 order from **Figure 10A** would not receive the maximal score, despite its appearing like a clear case of domino progression.  Regarding **10A and B** it would be clearer/more persuasive if the authors combined all of the interrupted progression/uninterrupted progression and compared those prevalences. For example, in 10A, display the prevalence of 1-2-3 and 2-1-3, compared to the interrupted pattern of 1-3-2. And in 10B, compare the uninterrupted 1-2-3-4, 2-1-3-4, 3-1-2-4, 4-1-2-3 with the others.
15. Typo in conclusion, page 19 first line – 'part part'.

Is the work clearly and accurately presented and does it cite the current literature?

Yes

If applicable, is the statistical analysis and its interpretation appropriate?

Partly

Are all the source data underlying the results available to ensure full reproducibility?

Yes

Is the study design appropriate and is the work technically sound?

Yes

Are the conclusions drawn adequately supported by the results?

Yes

Are sufficient details of methods and analysis provided to allow replication by others?

Yes

Reviewer Expertise:

DNA replication timing, genomics

We confirm that we have read this submission and believe that we have an appropriate level of expertise to confirm that it is of an acceptable scientific standard, however we have significant reservations, as outlined above.

# Response

**Published:** 2023-08-14

**Authors:** J Julian Blow

**Affiliations:** University of Dundee, UK

**Major points:**

- *The authors have not demonstrated why EdU immune-precipitation in early S phase cells provides higher resolution than Repli-seq of non-synchronized cells. Would similar wavelet analysis of Repli-seq data not also show similar trends? If the aim is to demonstrate that domains are replicon clusters, couldn't this be done by analyzing Repli-seq data without the need to synchronize the cells and pulldown with EdU?*
  The issue is a matter of the high degree of synchronisation required. Repli-seq provides only a snapshot of populations in early or late S phase, while our release from G1/S synchronization and detection of EdU incorporating regions at various time points gives us much more detailed dynamics. As shown in Figure 2 and Supplementary Figure S2 our data is highly consistent with previously published timing studies but shows the more detailed structure of cells as they progress through early S phase. As mentioned in the discussion, other ways of synchronisation could be used for future work.  
- *We are surprised not to see substantial progress along the cell cycle (i.e., PI intensity) even after 2 hours. Did the authors take any timepoints (for **Figure 1B/S1**) later than 2 hours? Could the authors add quantification (for example using gates) to show the progress along the x-axis in **Figure 1B**? The lack of progression raises a question about how representative the results are; do they reflect a normal unperturbed cell cycle or do they represent a specific case in which the entry into S phase is very slow.*
  During the time course, an increase in total cellular DNA content can be observed, but we agree it is less than might be expected. This is presumably a consequence of the synchronisation protocol – it is known that mimosine can cause double strand breaks, so we tried to minimise mimosine exposure. To reflect this, we have added a sentence to the Discussion: “Although we made attempts to limit it, our use of mimosine does create some double-strand breaks, which might reduce the total number of initiation events occurring at later stages.” We have also amended the text in the Results section to describe this: “This increase in DNA content can be seen in synchronised cells pulsed with EdU for 100-130 min (Figure 1b), where the total increase in DNA content is ~15%; shorter pulses of EdU (Underlying data: Supplementary Figure S1) also show increases in DNA content, with ~6% increase in DNA content after 40 min. This slightly slowed progression through S phase could be due to mimosine inducing a small number of double strand breaks (47-50) or reducing cellular dNTP pools (52).” Please also see response to Reviewer 3’s minor point 1.  

**Minor points:**

1. *In the second paragraph of the Introduction the authors state "Indeed, of the total number of potentially available replication origins present in diploid mammalian cells – roughly 100,000,000 – only approximately 10% will be used to initiate DNA replication". Is the 100,000,000 a typo? We are more familiar with lower estimates, specifically 500,000 licensed origins.*
  This was a consequence of us trying to be too generic and talking about mammalian cells. We have replaced with estimates about human diploid cells, and given the figure of 500,000.
2. *The source for the RT data is provided as https://www2.replicationdo-main.com/database.php, but this link is not accessible. As such the data is currently not accessible to the public.*
  We have now uploaded this data to the EBI site ( https://ftp.ebi.ac.uk/biostudies/fire/S-BSST/966/S-BSST966/Files/RT_U2OS_Bone.txt), as the replicationdomain site is not currently operational.
3. *The inset in Figure 1B is not explained.*
  In response to Reviewer 3’s minor point 1 we have replotted the FACS graphs in Figure 1 and removed the inset histograms .  
4. ***Figure 2C** - scale of graph missing (and explanation of the units of the score)*
  We have added a heatmap colour scheme to the Figure and added a sentence to the legend saying: “Dark blue represents a signal of zero or below, and red represents a signal of one.” Both chromosome position on the x axis and wavelet scale on the y axis are labelled.
5. **Reference 48** seems the wrong reference for page 9 (" *This is consistent with recent data on single DNA fibres which shows the high degree of stochasticity that occurs in origin firing*").
  We’ve now used the correct reference.
6. ***Figure 4A **and **B** – What does the 0-40 timepoint add over the 10-40 timepoint? Also why is the 10kb and the 50kb comparison important? Also, 4c and 4d come to justify the 70 percentile cutoff, but this has already been relied on in **Figure3**. Perhaps the figure order could be swapped between **Figures 3** and **4**? Or **4** could be moved to supplementary.*
  The reason for providing all the different analyses – both 10kb and 50 kb bins, and both 0-40 and 10-40 – is to show that the presence of a peak at around 500kb is quite robust to different data being used and how it is divided up. Since this is a major conclusion of the work, we think it best to show all the data. We think it is easier for readers to see the exemplar data in Fig 3 first, then see the numerical analysis in Figure 4, although following the text does require readers to go back and forth between them.
7. *When discussing **Figure 4D**, page 12, the authors say that the variance between the clusters is due to stochasticity between cells. We can think of 2 alternative explanations. Based on what we have observed in published data, and in our own lab's unpublished data, inter-origin distance has as much variance as the 20-fold variance shown in **Figure 4D**. Alternatively, the variance could reflect locus to locus variation in the time that different Replicon Cluster Domains become active. Although these three options are discussed in the discussion it might help to mention all three in the results as well, as the conclusion that stochasticity between cells is responsible remains not fully proven.*
  The text on page 12 discusses two possible explanations for the differences in peak height: cell-to-cell variation in origin density or cell-to-cell variation in the time that Replicon Cluster Domains become active in different cells. I think this latter explanation is equivalent to the locus to locus variation suggested by the reviewer.
  We have amended the text slightly to make this clearer and emphasise that in each case this would be due to cell-to-cell variation: “ *The difference in peak heights could be caused by a combination of two different effects: it could represent cell-to-cell variation in the densities of active origins in each Replicon Cluster Domain or it could represent some stochasticity in the time that Replicon Cluster Domains become active.*”
8. ***Figure 5B** – how the early replication domain are defined?*
  It was defined from the early timing domain data as illustrated in Figure 2b. The legend to Fig 5b has been amended to indicate this.
9. *Regarding the Isolated Replicon Cluster Domains:*
  1. ***Figures 7C-F** are based on only 72 isolated domains. How sure are they that the conclusions derived from the isolated domains are relevant to more crowded regions?*
    We can’t be totally sure because in more crowded regions the edges cannot be clearly determined. However, these crowded peaks do seem to grow in a similar manner – for example see Figure 8.
  2. *The authors say "We rejected from further analysis 51 peaks" was there a biological justification for removing these, instead of expanding the range of widths (e.g, 100-1500).*
    This was just because they exceeded the data limits we had set. Visual analysis suggested that these peaks were not being correctly fitted by the wavelet or Gaussian analysis due to either their unusual shape or being affected by other EdU incorporation impinging on the peaks, likely due to additional initiation events. This is likely related to point 3 by Reviewer 1 about timing transition regions and this reviewer’s point 11, but our data are not clear enough to unequivocally interpret these in terms of TTRs.
10. *Statistical tests are missing in **figures 7E-F, 9B-C** and **10**.*
  For Figure 7E-F we have added the missing statistical information to the legend. We have also added a new panel 7G which shows that the peak width increases are statistically significant. For Figure 9 we have added a new panel D which shows that the valley filling is statistically significant. For Figure 10 we have added a new panel E which shows that the positive value of the adjacency metric is highly significant. We also proved Standard Errors rather than Standard Deviations for Figures 7 and 9, as this better reflects our confidence in the mean values presented.
11. *In my understanding the edges of the timing domains reflect what is generally referred to as TTR. The authors should mention this, for example when discussing the valley filling in **Figure 8**, or in the discussion "As cells progress through S phase, the width of these peaks grows at rates consistent with the progression of a single pair of replication forks at the extreme edges of each peak.".*
  The ‘valleys’ analysed in Figure 8 are not TTRs as previously described, because TTRs are defined as being at the edges of individual Timing Domains, but the valleys we analyse are between two Replicon Cluster Domains that lie within an individual Timing Domain.  
12. *In **Figure 8**, the expansion of the bars should start from the 40-70m timepoint in line with what the authors wrote on page 14 ("The lack of significant growth between the first two time points …").*
  Because we wanted to stress that it is highly likely that further initiation events between the peaks is required, we wanted to show the most extreme example where the forks move outwards straight away. But the reviewer makes a good point that this movement is not clearly evident in the first time point.
  We have added the following sentence to the description of this data: “ *In addition, we showed in Figure 7 that fork-driven expansion of the edges of Replicon Cluster Domains is not clearly seen in the first time point due to the continued entry of cells into S phase.*”
13. *Near the end of the results the authors say "the mean spacing is 817 kb". This appears to be a mistake, based on **Figure 5**(0.817 is the number of peaks per Mbp. The real number should be 1.2 Mb spacing between peaks). The rest of the analysis of the adjacent Replicon Cluster Domains for **Figure 10** should be recalculated accordingly.*
  The wording in the text was unclear here and has been replaced by “Within individual timing domains, the mean distance between adjacent Replicon Cluster Domains is 817 kb (Figure 5b).” The rationale for the definition of ‘adjacent’ (which is somewhat arbitrary anyway) still stands.  
14. ***Figures 10C** and **D** use an elegant metric but do not accurately measure the domino effect, as they assume that the outermost origin must be the first one to be activated. For example, by this logic, the 2-1-3 order from **Figure 10A** would not receive the maximal score, despite its appearing like a clear case of domino progression.  Regarding **10A and B** it would be clearer/more persuasive if the authors combined all of the interrupted progression/uninterrupted progression and compared those prevalences. For example, in 10A, display the prevalence of 1-2-3 and 2-1-3, compared to the interrupted pattern of 1-3-2. And in 10B, compare the uninterrupted 1-2-3-4, 2-1-3-4, 3-1-2-4, 4-1-2-3 with the others.*
  This is one of the problems with using the ‘domino’ metaphor, as there is a possibility that the first domino in the stack is internal and should therefore fall in both directions. To indicate this possibility without confusing the reader, we have re-ordered the permutations in 10A and B so that in moving left to right it moves from less domino-like to more domino-like.
15. *Typo in conclusion, page 19 first line – 'part part'.*
  Corrected.

# Reviewer report: Reviewer response for version 1

**DOI:** 10.21956/wellcomeopenres.20781.r56117

**Published:** 2023-04-26

**Authors:** Ichiro Hiratani

**Affiliations:** 1 RIKEN Center for Biosystems Dynamics Research, Kobe, Japan

da Costa-Nunes JA et al., The location and development of Replicon Cluster Domains in early replicating DNA

 It has been generally accepted that metazoan genome replication proceeds by near synchronous firing of clusters of replication origins located adjacent to each other on the linear DNA sequence. However, the precise manner in which DNA replication proceeds within Mb-sized replication timing (RT) domains is not well understood.

 In this manuscript, the authors analyzed the earliest RT domains on the human genome by NGS and performed wavelet analysis followed by a series of in-depth data analyses to understand how DNA replication proceeds within these domains. Specifically, the authors synchronized human U2OS cells by thymidine block&release followed by an L-mimosine block&release to label the earliest replicating sequences by a 30-min EdU pulse. This was followed by biotinylation, pull-down by streptavidin beads, and NGS to identify the sites of EdU incorporation on the U2OS genome. A series of experiments were performed to analyze 10–40, 40–70, 70–100, 100–130, and 0–130 min time points after release from the L-mimosine block.

 The authors made the following findings:

1. The most prominent component of the early replicating signal was at the size of ~500 kb, which they defined as the Replicon Cluster Domains (RCDs).
2. RCDs were separated by ~1.2 Mb on average, and the number of RCDs was proportional to the size of the RT domains.
3. The >20-fold variation in replication peak height among RCDs was explained by the variation in the activation timing of RCDs rather than the difference in active origin density of RCDs.
4. RCDs gradually broadened in width over time at a pace consistent with the progression of two flanking forks at both ends of a given RCD proceeding at 1–1.5 kb/min.
5. The authors' data analysis suggested that most RCDs are still replicating internally even at the last time point, in parallel with the broadening of RCDs at both flanks by outward-moving forks.
6. They also addressed whether multiple RCDs within large early RT domains completed the replication of these domains by simple fusion of outward-moving forks flanking RCDs or by new initiation events within the 'valley' regions between RCD peaks. The authors' analysis suggested the latter, with later activating RCDs present in the 'valley regions' between early activating RCD peaks.
7. Finally, the authors tested the so-called 'domino' activation model of replication foci within single early RT domains. Their analyses suggested that adjacent RCDs tend to be activated much more frequently than by chance, which supported the 'domino' activation model.

It appeared to me that the analyses were done carefully and thoroughly to provide evidence supporting the points made by the authors. It is impressive to see how such simple NGS experiments can provide valuable insights into the regulation of DNA replication inside RT domains and underscores the importance of exploiting large-scale data sets through in-depth analyses. I am positive about the manuscript. I do have a few comments, though.

1. U2OS cells are aneuploid. Figure S2 G1 data indeed shows various copy number variations. I wonder if this could be a potential confounding factor in interpreting DNA replication data for some genomic regions.
2. A recent paper by the authors using optical mapping technology (Ref. 7) did not support the 'domino' activation model of replication initiation zones (IZ) and instead supported the stochastic activation model for neighboring IZs. It would help the readers if the authors discussed these differences and provided potential interpretations for the discrepancy.
3. It is interesting that while RCDs are gradually broadening in width at a pace consistent with the progression of two outward-moving forks at their flanks, most RCDs are still replicating internally even at the last time point of the experiment performed. Eventually, however, a time should come after which only the two outward-moving forks at the RCD flanks are synthesizing DNA in the absence of replication activities inside the RCD (as in Fig. 7a, the very bottom cartoon), i.e., timing transition region (TTR) replication phase. I wonder why the authors did not observe such a phase (as in Fig. 7a at the very bottom) in their experiment. For instance, is this due to variability in the timing of entry into the S-phase among cells after release from the L-mimosine block? Was 130 min not sufficient for the earliest replicating RCDs (replication foci) to complete their replication?
4. If the level of synchrony was sufficient, it seems possible to witness such a phase (as in Fig. 7a at the very bottom), especially for small early RT domains with only a single RCD.
5. (Optional) If the authors could see this, I am very curious as to the genomic locations of the two outward-moving forks at the RCD flanks in such phase based on the authors' analysis and how this is related to the TAD boundary positions because the authors previously reported the alignment of early borders of TTRs to TAD boundary positions. This would provide valuable insights into the 3D organization of TTRs and the relationship to replication activities. Hi-C data for U2OS cells are available (see links below), so this analysis seems feasible without additional experiments. Also, this might allow the authors to relate RCDs to intra-TAD structures.
https://www.ncbi.nlm.nih.gov/geo/query/acc.cgi?acc=GSM6552772
https://www.ncbi.nlm.nih.gov/geo/query/acc.cgi?acc=GSM4194463
https://www.ncbi.nlm.nih.gov/geo/query/acc.cgi?acc=GSM4194464

**Minor points:**

- Page 9, Ref.48 seems incorrect > a different Wang et al., paper (Mol Cell, 2021) and the same as Ref. 7, I believe.
- Figure 7b, I did not understand the figure very well. The vertical axis probably represents replication signal intensity (which should be described in the figure), but how could the height of the blue-green dashed line be identical to that of the blue line?
- Figure 8, I would like to see population Repli-seq data (as in Fig. 2b) for comparison to see that the valleys between peaks are part of the same early replicating RT domain.
- Page 14 & Figure 5a, how did the authors define the distance between adjacent RCDs? My prediction is end-to-end distance based on how things are described on page 14, but it could be the peak-to-peak distance between two adjacent RCDs. This should be described.
- Page 18, Although there was substantial a variability in > Although there was substantial variability in (typo)
- Page 19, line 1: this is at least in part part caused by > this is at least in part caused by

Is the work clearly and accurately presented and does it cite the current literature?

Yes

If applicable, is the statistical analysis and its interpretation appropriate?

I cannot comment. A qualified statistician is required.

Are all the source data underlying the results available to ensure full reproducibility?

Yes

Is the study design appropriate and is the work technically sound?

Yes

Are the conclusions drawn adequately supported by the results?

Yes

Are sufficient details of methods and analysis provided to allow replication by others?

Yes

Reviewer Expertise:

NA

I confirm that I have read this submission and believe that I have an appropriate level of expertise to confirm that it is of an acceptable scientific standard, however I have significant reservations, as outlined above.

# Response

**Published:** 2023-08-14

**Authors:** J Julian Blow

**Affiliations:** University of Dundee, UK

**Main Point 1:** “U2OS cells are aneuploid. Figure S2 G1 data indeed shows various copy number variations. I wonder if this could be a potential confounding factor in interpreting DNA replication data for some genomic regions.”  

 This is indeed a possibility but is unlikely to have a significant effect on our interpretations. It would only affect the wavelet analyses in the rare cases where a translocation in the U2OS genome occurs within one of the 500 kb wavelet peaks. We have added the following text to page 12: “ *It should be noted that our sequences have been mapped onto a normal human genome, but since U2OS have a number of chromosome rearrangements and copy number variations this could be a potential confounding factor in interpreting DNA replication data for a few genomic regions.*”

**Main Point 2:** “ *A recent paper by the authors using optical mapping technology (Ref. 7) did not support the 'domino' activation model of replication initiation zones (IZ) and instead supported the stochastic activation model for neighboring IZs. It would help the readers if the authors discussed these differences and provided potential interpretations for the discrepancy.*”

 The term "domino model” has been invoked to mean at least two different things. In the way we have used the term, it simply refers to the descriptive observation that nearby structural domains observed cytologically tend to fire sequentially - in other words, replication foci labeled with pulse - 60 minute chase (to complete the first foci) - second pulse -  often are genetically close to each other. The prior optical replication mapping (ORM) paper (Wang et al, 2021) to which the reviewer refers could not address this version of the domino model because ORM is a single pulse label that cannot measure sequential events.

 Moreover, the fibres in ORM average ~350kb, so they could not contain two adjacent domains. We acknowledge that there is a sentence in the ORM paper with the term “domino model” in it, but this was referring to a more strict version of the domino model in which a mechanism is invoked that forks emanating from one domain invade the adjacent domain activating initiation. This would create adjacent IZs of different sizes on the same single fibres (fork encroaching and stimulating initiation nearby) and we did not observe that. In fact, this mechanism was ruled out by Dimitrova and Gilbert, NCB 2000, who showed that by disabling the S phase checkpoint, one could get sequential foci firing in the proper order while completely inhibiting fork elongation.

 To address this confusion in the paper, we have added to the sequential activation sub-section of the results the following clarification: “ *The mechanism by which this occurs is not known, however, it is unlikely to occur by the forks emanating from one domain stimulating initiation of the adjacent domain because: a) disabling the S phase checkpoint in the absence of processive elongation of replication forks still results in the sequential firing of replication foci in the proper order (Dimitrova and Gilbert, NCB, 2000) and; b) optical replication mapping studies failed to find evidence for new bursts of DNA synthesis near longer tracks (Klein et. al., Science, 2021).*”

**Main Points 3 and 4**: “ *It is interesting that while RCDs are gradually broadening in width at a pace consistent with the progression of two outward-moving forks at their flanks, most RCDs are still replicating internally even at the last time point of the experiment performed. Eventually, however, a time should come after which only the two outward-moving forks at the RCD flanks are synthesizing DNA in the absence of replication activities inside the RCD (as in Fig. 7a, the very bottom cartoon), i.e., timing transition region (TTR) replication phase. I wonder why the authors did not observe such a phase (as in Fig. 7a at the very bottom) in their experiment. For instance, is this due to variability in the timing of entry into the S-phase among cells after release from the L-mimosine block? Was 130 min not sufficient for the earliest replicating RCDs (replication foci) to complete their replication? If the level of synchrony was sufficient, it seems possible to witness such a phase (as in Fig. 7a at the very bottom), especially for small early RT domains with only a single RCD*.”

 We did look for TTRs as suggested and also considered small early RT domains. As the reviewer suggests, this was confounded by the fact that the synchrony decays over the period of the analysis. Although our data would be consistent with TTRs, we decided not to try to analyse these as the data were too ambiguous.  

**Main Point 5**. “ *If the authors could see this, I am very curious as to the genomic locations of the two outward-moving forks at the RCD flanks in such phase based on the authors' analysis and how this is related to the TAD boundary positions because the authors previously reported the alignment of early borders of TTRs to TAD boundary positions. This would provide valuable insights into the 3D organization of TTRs and the relationship to replication activities. Hi-C data for U2OS cells are available (see links below), so this analysis seems feasible without additional experiments. Also, this might allow the authors to relate RCDs to intra-TAD structures.*”

 We examined whether the ‘isolated’ RCDs analysed in Figures 6 and 7, where the RCD boundaries are fairly well defined, might correspond to published TAD boundaries as suggested. Despite some peaks reaching high association scores with overlapping TADs, none of these scores was found to be statistically significant at a 0.05 false discovery rate (FDR) level.  

**Minor points:**

- *Page 9, Ref.48 seems incorrect > a different Wang et al., paper (Mol Cell, 2021) and the same as Ref. 7, I believe.*
  We’ve now used the correct reference.
- *Figure 7b, I did not understand the figure very well. The vertical axis probably represents replication signal intensity (which should be described in the figure), but how could the height of the blue-green dashed line be identical to that of the blue line?*
  We have clarified the description of the cartoon in the figure legend. We also appreciate the point that the blue-green dashed line should be higher than the blue or green lines, so have modified the figure accordingly.

- *Figure 8, I would like to see population Repli-seq data (as in Fig. 2b) for comparison to see that the valleys between peaks are part of the same early replicating RT domain.*
  We have added the Repli-Seq (timing domain) data to the figure as requested. We appreciate this suggestion as one of the selections (chromosome 3, 111-121 MbP) spanned two timing domains, so we have replaced this with a new selection (chromosome 7 39-49 Mbp).
- *Page 14 & Figure 5a, how did the authors define the distance between adjacent RCDs? My prediction is end-to-end distance based on how things are described on page 14, but it could be the peak-to-peak distance between two adjacent RCDs. This should be described.*
  The distance was between the peaks as defined by the 500 kb wavelet analysis; the text has been amended to clarify this (“Figure 5a shows that the peak separation…”).
- Page 18, Although there was substantial a variability in > Although there was substantial variability in (typo)
  Corrected.
- Page 19, line 1: this is at least in part part caused by > this is at least in part caused by
  Corrected.
